# An Advanced Mechanically Active Osteoarthritis‐on‐Chip Model to Test Injectable Therapeutic Formulations: The SYN321 Case Study

**DOI:** 10.1002/adhm.202401187

**Published:** 2024-09-24

**Authors:** Cecilia Palma, Stefano Piazza, Roberta Visone, Rune Ringom, Ulf Björklund, Antonio Bermejo Gómez, Marco Rasponi, Paola Occhetta

**Affiliations:** ^1^ Department of Electronics Information and Bioengineering Politecnico di Milano Via Ponzio 34/5 Milan 20133 Italy; ^2^ BiomimX Srl Viale Decumano 41 MIND – Milano Innovation District Milan 20157 Italy; ^3^ Recipharm OT Chemistry AB Virdings allé 18 Uppsala 754 50 Sweden; ^4^ UB‐consulting AB Trädgårdsgatan 7A Uppsala 753 09 Sweden; ^5^ Synartro AB Dalgatan 16A Uppsala 752 18 Sweden

**Keywords:** cartilage, organ‐on‐chip, osteoarthritis, therapy

## Abstract

Current treatments for osteoarthritis (OA) often fail to address the underlying pathophysiology and may have systemic side effects, particularly associated with long‐term use of non‐steroidal anti‐inflammatory drugs (NSAIDs). Thus, researchers are currently directing their efforts toward innovative polymer‐drug combinations, such as mixtures of hyaluronic acid viscoelastic hydrogels and NSAIDs like diclofenac, to ensure sustained release of the NSAID within the joint following intra‐articular injection. However, the progress of novel injectable therapies for OA is hindered by the absence of preclinical models that accurately represent the pathology of the disease. The uBeat® MultiCompress platform is here presented as a novel approach for studying anti‐OA injectable therapeutics on human mechanically‐damaged OA cartilage microtissues, in a physiologically relevant environment. This platform can accommodate injectable therapeutic formulations and is successfully tested with SYN321, a novel diclofenac‐sodium hyaluronate conjugate under development as a treatment for knee OA. Results indicate the platform's effectiveness in evaluating therapeutic potential, showing downregulation of inflammatory markers and reduction in matrix degradation in OA cartilage micro‐tissues treated with SYN321. The uBeat® MultiCompress platform thus represents a valuable tool for OA research, offering a bridge between traditional in vitro studies and potential clinical applications, with implications for future drug discovery.

## Graphical abstract

The uBeat® MultiCompress platform offers a novel approach for studying injectable therapies on human osteoarthritic (OA) cartilage microtissues in a physiologically‐ and mechanically‐relevant environment. It can accommodate therapeutic formulations like SYN321, a promising diclofenac‐sodium hyaluronate conjugate for knee OA treatment. Results demonstrate its effectiveness in evaluating therapeutic potential, bridging the gap between in vitro research and clinical applications.

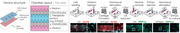

## 1 Introduction

Osteoarthritis (OA) is the most common degenerative joint disorder and a leading cause of joint pain and disability, strongly impacting patients’ quality of life and consuming a significant portion of healthcare resources worldwide.^[^ ^1^, ^2^ ^]^ Moreover, OA prevalence is in continuous growth, with a recent study that revealed that OA cases more than doubled in nearly 30 years globally, reaching 527.81 million cases in 2019, of whom 365 categorized as knee OA.^[^ ^3^ ^]^ Specifically, OA is characterized by a failure of the entire synovial joint, with a strong involvement of articular cartilage, that undergoes matrix breakdown, enhanced synthesis of extracellular matrix (ECM)‐degrading enzymes, as well as hypertrophic‐like maturation, due to excessive mechanical loadings and catabolic components.^[^ ^4^, ^5^, ^6^ ^]^

Therapeutic agents that are generally used to relieve OA pain and inflammation include analgesics, glucocorticoids and nonsteroidal anti‐inflammatory drugs (NSAIDs).^[^ ^7^ ^]^ Among those treatments, NSAIDs (e.g., ibuprofen, naproxen, celecoxib and diclofenac),^[^ ^8^ ^]^ are the most commonly employed therapies and their mechanism of action involves the inhibition of cyclooxygenases (COX‐1 and COX‐2) preventing the biosynthesis of prostaglandins, that induce inflammation and pain.^[^ ^9^ ^]^ However, NSAIDs administration presents several challenges. Oral administration of NSAIDs results in moderate pain reduction and enhanced physical functions in OA patients, however long‐term therapy has been associated with various systemic side effects, such as gastric ulcers and bleeding, cardiovascular complications, as well as renal dysfunctions.^[^ ^10^, ^11^ ^]^ Intra‐articular (IA) administration of NSAIDs offers the advantage of delivering targeted pain relief and reducing inflammation directly within the affected joint, avoiding adverse systemic effects.^[^ ^12^ ^]^ Nonetheless, low‐molecular‐weight molecules, e.g. NSAIDs, injected directly in the articulation are rapidly cleared from synovial fluid. This results in the need for frequent and repeated injections increasing risk of infections and economic burden, making this solution less viable in contemporary medical practices.^[^ ^12^, ^13^ ^]^ Consequently, researchers’ focus is on developing innovative polymer‐drug conjugates (e.g., combining viscoelastic hydrogels like hyaluronic acid (HA) and NSAIDs such as diclofenac^[^ ^12^, ^13^, ^14^, ^15^, ^16^ ^]^) able to guarantee NSAID sustained release into the joint after IA injection as an alternative to oral treatments. This would allow the use of lower amounts of the NSAID, minimizing systemic side‐effects associated to oral treatments, and providing a robust and sustained pain relief. To this aim, Synartro AB has developed Hydro‐link, a proprietary linker that enables an efficient slow release of local injectable compounds, driving durable treatment effects at very low dosage that can potentially be beneficial in medical needs that require local slow release of compounds, with safety concerns related to systemic exposure. The platform's slow release is mediated through the chemical structure of Hydro‐link, a hyaluronan derivative, which contains specific ester groups that easily form bonds between different types of molecules. These ester groups are slowly hydrolyzed upon contact with biological fluids (non‐enzymatic and/or enzymatic hydrolysis), driving slow release of the active compounds and consequently durable treatment effect. The Hydro‐link has been integrated in SYN321, a novel IA investigational drug based on diclofenac linked to a modified sodium hyaluronate (NaHA) backbone, recently developed by Synartro AB as a treatment for knee OA pain.^[^ ^13^ ^]^

Although promising, the development of such novel anti‐OA injectable therapeutic solutions (e.g., SYN321) is today still hampered by the lack of preclinical models representative of the human pathology. To date, therapeutics are indeed mainly screened exploiting animal testing, by means of either models characterized by naturally occurring primary OA or by chemically/surgically induced secondary OA.^[^ ^17^ ^]^ A major example is represented by the monoiodoacetate (MIA) model in rats and mice, where the intra‐articular injection of MIA into a knee joint induces OA‐like lesions, functional impairments and pain.^[^ ^18^, ^19^ ^]^ However, animal models exhibit manifold limitations, such as high inter‐species variability, poor translatability of drug testing outcomes, and limited possibility to gain insight into disease and drug mechanisms of action at the cellular and subcellular levels.^[^ ^20^, ^21^ ^]^ Moreover, human joints are unique in their structure and load‐bearing patterns, which are often not fully replicated in the anatomy and physiology of animal models.^[^ ^22^ ^]^ As mechanical stress and joint loading play significant roles in OA onset and progression,^[^ ^23^ ^]^ differences in joint articular biomechanics between humans and animals are particularly relevant in OA research. Thus, there is an increasing need for developing innovative in vitro human OA models, such as Organs‐on‐Chip (OoC), to better understand the mechanisms of combined therapeutic products in a physiologically and biomechanically relevant environment.

Over the past years, some OoC models have emerged to in vitro reproduce and investigate cartilage pathophysiology and biomechanics.^[^ ^24^, ^25^ ^]^ For example, Lee et al. developed a pneumatic microfluidic device to compress chondrocytes in alginate hydrogel constructs with different magnitudes, aiming at studying their mechanobiology.^[^ ^26^ ^]^ In another study, Paggi et al. designed a monolithic polydimethylsiloxane (PDMS)‐based platform to exert multi‐modal and multi‐axial mechanical cues on chondrocytes embedded in agarose matrix, to assess the influence of mechanical stimulation on chondrocyte behavior.^[^ ^27^ ^]^ In a previous work reported by our research group, a microfluidic platform was developed to recapitulate 3D articular cartilage microtissues by culturing human articular chondrocytes embedded in a Poly(ethylene glycol) (PEG) hydrogel, and to induce OA traits upon application of strain‐controlled hyperphysiological compression (HPC), i.e., 30%. The microphysiological model could be successfully exploited to reproduce chondrocytes catabolism and hypertrophy in OA, as well as to test the efficacy of soluble NSAIDs.^[^ ^28^ ^]^ However, to the best of our knowledge, no OA cartilage microphysiological models have been yet developed, allowing the integration of injectable therapeutic formulations in a physiologically and biomechanically relevant joint environment.

In this work, we present uBeat MultiCompress Platform, a novel microfluidic platform aimed at recapitulating OA‐like 3D cartilage microtissues (namely uKnee model), that offers the possibility to test injectable therapeutic formulations in direct contact with cell microtissues, both subjected to the same native‐like mechanically active environment. The platform was qualified with Supartz, a commercially available IA‐HA‐based product.^[^ ^29^ ^]^ Upon qualification, the model was applied to test the efficacy of SYN321, demonstrating its potentiality to identify molecular and cellular mechanisms of action and to further dissect the effect of a new therapeutic option in relieving OA symptoms.^[^ ^13^ ^]^

## 2 Results

### 2.1 uBeat^®^ MultiCompress: A Microfluidic Platform for the Co‐Culture of Cartilage Microtissues and Injectable Therapeutic Formulations

**Figure 1:**
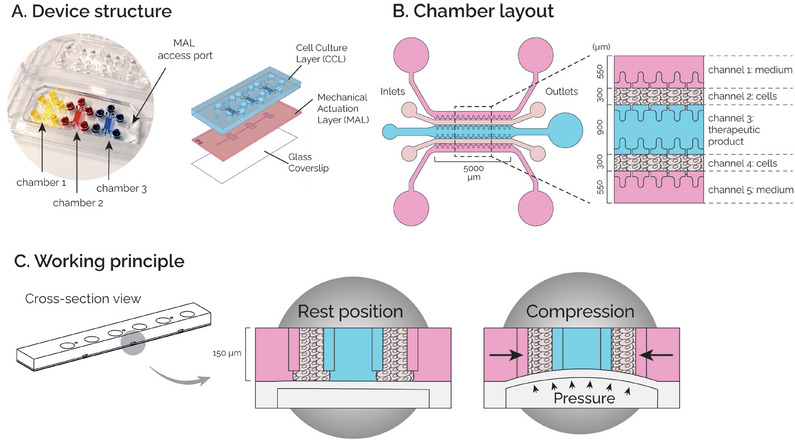
A) Device structure: the device comprises three biologically independent chamber and it is composed of three superimposed layers: a Cell Culture Layer (CCL), a Mechanical Actuation Layer (MAL), and a Glass Coverslip. B) Chamber layout: Each culture chamber is composed of five channels, i.e. two outermost channels for culture medium (channels 1 and 5), two for cell‐laden hydrogels (channels 2 and 4), and one for the injectable therapeutic product (channel 3). C) Working principle. At rest position, cell‐laden hydrogels and the therapeutic product are not mechanically stimulated. Upon pressurization of the MAL, they are subjected to a confined mechanical compression (30%).

A microscale cell culture platform, namely uBeat MultiCompress, was conceived to apply a uniform and confined mechanical compression to a complex 3D structure, composed of an injectable therapeutic formulation interposed within two cartilage microconstructs. The device design was optimized starting from a previous platform, aimed at recapitulating 3D OA cartilage micro‐tissues through HPC,^[^ ^28^ ^]^ and adapted to host injectable therapeutic formulations. Specifically, the proposed device is composed of three layers: i) the Cell Culture Layer (CCL), divided by a flexible membrane from ii) the Mechanical Actuation Layer (MAL), and iii) a glass coverslip (**Figure** **1**A). In details, the CCL comprises three cell culture chambers, each composed by 5 channels (Figure 1B), i.e., two channels delimited by rows of T‐shaped overhanging posts conceived to host 3D cartilage micro‐constructs (i.e., channel 2 and 4), a central channel for therapeutic product injection (i.e., channel 3), and the two outermost channels for culture medium supply (i.e., channel 1 and 5). The presence of the MAL allows to apply a HPC to the entire microstructure by exploiting the uBeat technology (BiomimX Srl). In details, in the rest position, a gap separates the overhanging posts from the underneath flexible membrane, maintaining the 3D microconstructs and the therapeutic product in an undeformed state. Upon pressurization of the MAL, the flexible membrane bends upwards until it abuts against the posts’ base, causing confined compression of the entire microstructure thanks to the posts’ shape and dimensions (Figure 1C).^[^ ^28^ ^]^ Notably, the gap underneath the hanging posts (i.e., 43 µm) defines the strain level of mechanical compression,^[^ ^30^ ^]^ here allowing to apply a 30% HPC to cartilage micro‐constructs, together with the therapeutic product.

### 2.2 uBeat^®^ MultiCompress Mechanical Characterization

uBeat MultiCompress was tested to verify that the constructs seeded in the CCL were subjected to a confined mechanical compression upon pressurization of the MAL, and to assess whether the strain field in each channel was in accordance with the values computed both numerically and experimentally in the previous single‐channel version of the platform.^[^ ^28^ ^]^

**Figure 2:**
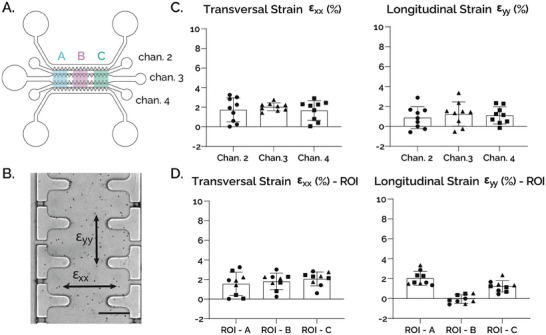
A) Layout of the chamber with highlighted ROIs and channels considered for the characterization. B) Top view of channel 3 (scalebar 100 µm). C) Results in both x and y directions are consistent with target strain obtained in the previous version of the device. Transversal strain: (ch.2) 1.73 ± 1.23%, (ch.3) 2.04 ± 0.41%, (ch.4) 1.67 ± 1.00%; Longitudinal strain: (ch.2) 0.88 ± 1.11%, (ch.3) 1.24 ± 1.22%, (ch.4) 1.11 ± 0.86% [n=9]. D) Results of Transversal strain in each ROI – (A) 1.57± 1.19%, (B) 1.81 ± 0.85%, (C) 2.07 ± 0.71%, and Longitudinal strain in each ROI – (A) 2.04 ± 0.70%, (B) ‐0.05 ± 0.42%, (C) 1.23 ± 0.57%.

The strain field within the channels of the CCL was specifically measured. In detail, fibrin gel loaded with polystyrene microbeads was injected in channels 2, 3, and 4 while medium channels and reservoirs were filled with Phosphate‐buffered Saline (PBS) (**Figure** **2**A). Upon application of HPC, strain field was estimated from microbeads’ displacement along transversal (x) and longitudinal (y) directions (Figure 2B). Strain values of 1.73 ± 1.23% (x direction) and 0.88 ± 1.11% (y direction) for channel 2, 2.04 ± 0.41% (x direction) and 1.24 ± 1.22% (y direction) for channel 3 and 1.67 ± 1.00% (x direction) and 1.11 ± 0.86% (y direction) for channel 4 were obtained (Figure 2C). We then computed transversal and longitudinal strains in three different regions of interest (ROIs) along the same device's longitudinal axis (i.e., ROI A, B, and C in Figure 2A) to investigate the position‐dependent strain distribution. While no differences were detectable in transversal strains (x direction), lower values of longitudinal strains (y direction) could be observed in the central area of the device (i.e., −0.05 ± 0.42% in ROI B) as compared to the peripheral areas (i.e., 2.04 ± 0.70% and 1.23 ± 0.57% in ROIs A and C, respectively) (Figure 2D).

Overall, results were in good accordance with experimental and computational values measured in the previous version of the device^[^ ^28^ ^]^ and confirmed that a confined compression state was reached in every channel of uBeat MultiCompress, with negligible transversal and longitudinal strains (i.e., more than one order of magnitude lower than the 30% confined compression applied along the z‐axis).

### 2.3 uBeat^®^ MultiCompress Compatibility with Viscous Therapeutic Products

uBeat MultiCompress was specifically designed to enable the injection of viscous therapeutic formulations within complex 3D cell microconstructs. Introducing an additional channel for the loading of the therapeutic product, implies that the product can be injected into the designed channel at any time during the culture, while staying confined between the two cartilage microtissues during injection. The compliance to this requirement is crucial since many of the anti‐OA therapeutic products are hydrogels (e.g., HA‐based solutions, Figure S1, Supporting Information) with medium‐to‐high viscosity, thus exhibiting a high resistance when injected in micro‐channels. To verify this specific functionality of the device, the success rate of injection in the therapeutic product channel (i.e., channel 3, Figure 1B) was assessed by testing three products: SYN321 (provided by Synartro AB), sodium hyaluronate (NaHA, 15 mg mL^−1^, purchased from HTL Biotechnology with an intrinsic viscosity of 1.54 m^3^ kg^−1^) and Supartz (Seikagaku Corporation). Despite the high viscosity, all the substances could be aspirated and handled with a pipette, and injection in channel 3 was achieved with a succession rate of 100% for Supartz and SYN321 and of 89% for NaHA (as reported in **Table** **1**, together with molecular weights).

**Table 1:** Results of injection tests on both commercially available and under development therapeutic formulations. For each gel, 3 chips were used (n = 9 injections). ^1^Molar mass calculated by asymmetrical flow field‐flow fractionation (AF4) analysis; ^2^Altman et al. Cartilage (2016).

Substance name	Average molecular weight [kDa]	Success rate
SYN321	611^1^	100%
NaHA	667^1^	89%
Supartz^®^	900^2^	100%

### 2.4 Qualification of the uBeat^®^ MultiCompress with a Commercially Available Therapeutic Product

**Figure 3:**
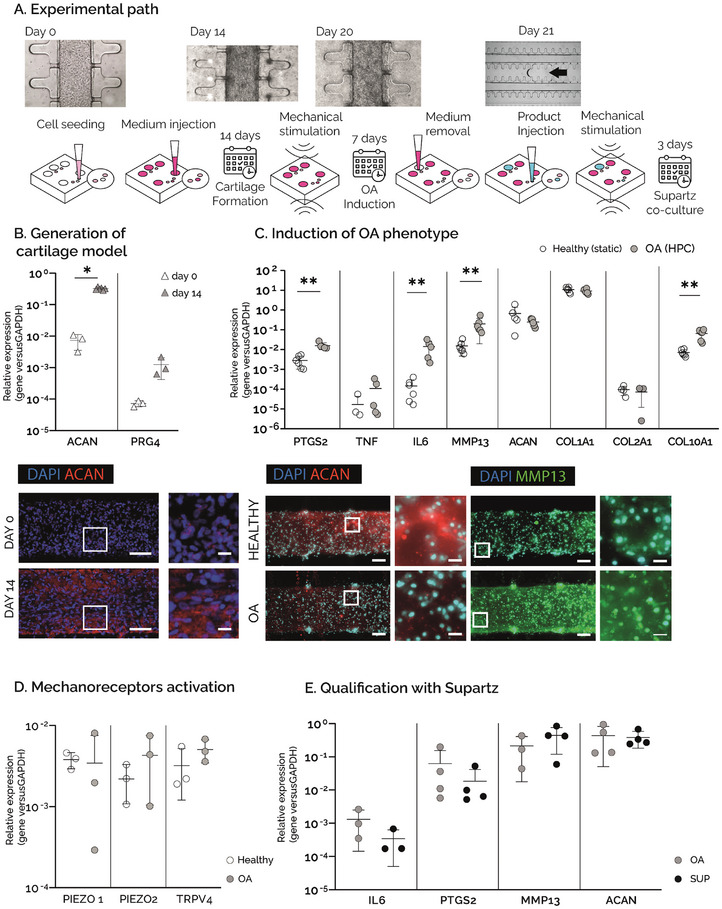
A) Experimental path: hACs embedded in fibrin gel were cultured in the uBeat® MultiCompress Platform for 14 days to achieve mature cartilage constructs. A HPC was then applied for 7 days to induce OA traits. Culture medium was then removed from the central channel of the culture chamber, followed by the injection of Supartz®, that was co‐cultured with OA cartilage micro‐tissues for 3 days under mechanical stimulation. B) Generation of cartilage model. Gene expression analysis of *ACAN* and *PRG4* at day 0 (n=3) and day 14 (n=6, *p<0.05), and immunofluorescence staining of nuclei (blue) and aggrecan (in red) at day 0 and day 14. Scale bar =100µm, scale bar magnfied pictures = 20 µm. C) Induction of OA phenotype. Gene expression analysis performed on cartilage constructs at day 21, cultured either in static condition (healthy) or under HPC (OA). **p<0.01, *p<0.05. Fluorescence stainings of nuclei (in light blue), aggrecan (in red), and MMP13 (in green) in healthy and OA samples. Scale bar = 100µm, scale bar magnfied pictures = 20 µm. D) Activation of mechanoreceptors. Gene expression analysis performed on cartilage constructs at day 21, cultured either in static condition (healthy) or under HPC (OA). **p<0.01, *p<0.05. E) Qualification with Supartz®. Gene expression analysis performed on cartilage constructs at day 23, comparing mechanically stimulated devices without Supartz® (OA) and mechanically stimulated devices with Supartz® (SUP). n=4 for each condition.

uBeat MultiCompress was first used to generate a healthy cartilage model, subsequently guided towards an OA phenotype through the application of HPC, to confirm the results obtained in the previous version of the platform.^[^ ^28^ ^]^ Supartz, a commercially available HA that already proved anti‐inflammatory effects on OA cartilage,^[^ ^29^ ^]^ was then used to qualify the platform (**Figure** **3**A).

Specifically, commercially human articular primary chondrocytes (hACs) embedded in fibrin gel were cultured in uBeat MultiCompress for 14 days in static conditions, obtaining healthy cartilage microtissues as demonstrated by a significant upregulation of *ACAN* and *PRG4* expression at gene level at day 14 compared to day 0. The result was also confirmed at protein level as shown by immunofluorescence analysis, revealing a high deposition of aggrecan in the ECM (Figure 3B). After this, the cartilage microtissues were subjected to a 30% cyclic HPC for 7 days. The mechanical stimulation was provided through BiomimX control system uBoX, using a pattern that resembles the daily walk (i.e., frequency of 1 Hz, 2 h on – 4 h off – 2 h on – 16 h off). Healthy control platforms were kept in culture in static conditions for the whole stimulation period. The induction of an HPC‐driven OA phenotype in the model was demonstrated by the upregulation of genes related to inflammatory pathways (Figure 3C). Specifically, *PTGS2*, a gene encoding for the enzyme COX‐2, and *IL6* were significantly upregulated in the OA samples with respect to controls, while the pro‐inflammatory gene *TNF* showed an increasing trend. HPC also caused a significant increase in *MMP13* expression and a decreasing trend in *ACAN* expression. This enhancement of matrix degradation in OA condition was also confirmed by immunofluorescence analysis, as evidenced by a decreased expression of aggrecan in the ECM and an increased intracellular expression of MMP13 in the OA microtissues. While no significant changes were detected for *COL1A1* nor *COL2A1* expression at gene level, HPC caused a significantly higher expression of *COL10A1*, evidencing a shift of cartilage phenotype towards hypertrophy. Moreover, as shown in Figure 3D, mechanical overload induced the upregulation of *PIEZO2* and *TRPV4*, encoding for the mechanoreceptors Piezo2 and transient receptor potential vanilloid TRPV4, respectively. On the other hand, *PIEZO1* expression appeared not to be modulated after 7 days of HPC compression, accounting for a high variability especially in the stimulated OA condition.

After the generation of an OA model, Supartz was successfully injected in all the devices and cultured for three days under dynamic conditions. As shown in Figure 3E, the expression of inflammatory cytokines *IL6* and *PTGS2* was lower in Supartz‐treated samples as compared to OA controls. Expression of matrix‐related genes *MMP13* and *ACAN* was conversely not modulated in Supartz‐treated samples as compared to OA controls.

Overall, the uBeat MultiCompress platform was successfully used to generate a mechanically induced OA model that was qualified with a commercial product, Supartz, which confirmed to have an anti‐inflammatory effect^[^ ^31^ ^]^ while no matrix‐related effect on pathological cartilage microtissues.

### 2.5 A Case Study: SYN321 as Novel Investigational Drug to Treat Symptomatic OA

**Figure 4:**
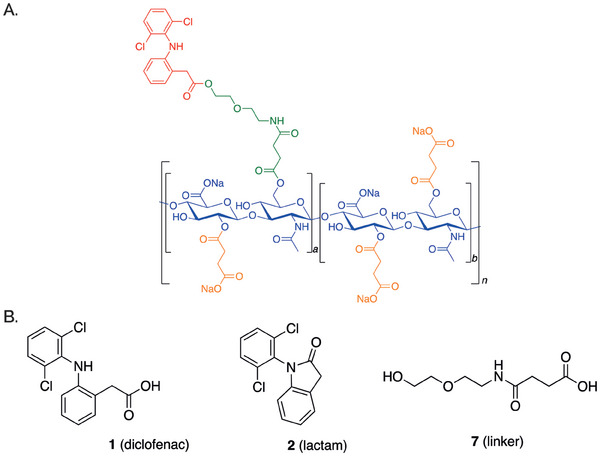
SYN321. A) The structure of SYN321 includes four components, i.e. diclofenac (in red), NaHA (in blue), a 3‐{[2‐(2‐hydroxyethoxy)ethyl] carbamoyl} propanoic ester molecule (‘linker’, in green) and a succinic ester (in orange). B) Structure of main metabolites upon SYN321 hydrolysis in human plasma and synovial fluid, i.e. diclofenac, diclofenac lactam, and linker.

As introduced above, SYN321 represents a pioneering IA drug candidate tailored for OA therapy, combining a hyaluronan backbone linked to diclofenac via a compact linker (diclofenac ethoxyethylamino succinylhyaluronan, as illustrated in **Figure** **4**A). The formulation is engineered to ensure effective and sustained pain relief, merging diclofenac's robust anti‐inflammatory properties alongside the lubricating effects of hyaluronan. In details, the structure of SYN321 includes four components: diclofenac (red part in Figure 4A), NaHA (blue part in Figure 4A), a 3‐{[2‐(2‐hydroxyethoxy)ethyl] carbamoyl} propanoic ester molecule (abbreviated “linker”; green part in Figure 4A) and a succinic ester (orange part in Figure 4A). The linker can be attached to either of the two most reactive alcohol groups of the hyaluronan molecule (position 2 of the glucuronic acid and position 6 of the glucosamine). A mixture of both is the most realistic final structure of SYN321. For simplification the linker in Figure 4A has been drawn in the glucosamine moiety (a = 1, b = 5).

### 2.6 Hydrolysis Profile of SYN321 and Diclofenac Release of in Human Plasma and Synovial Fluid

The hydrolysis profile and release of both diclofenac and linker of SYN321 was studied over a 48‐h period at 37 °C both in human plasma and in synovial fluid, with a starting SYN321 concentration of 1 µg mL^−1^. Three main metabolites were identified: compound 1 (i.e., diclofenac) and compound 2 (i.e., diclofenac lactam) result from the cleavage of the ester bond of the diclofenac moiety, and compound 3 (i.e., linker) is generated after the cleavage of the two ester bonds connecting the linker to diclofenac and hyaluronan (Figure 4B).

In human plasma (**Table** **2**, entries 1–5), diclofenac (1) was initially detected, but quantification was only possible after 4 h (11.4 nM), with the concentration increasing over time (125 nM in 48 h). Its amount constituted 67% of the total initial diclofenac bonded to SYN321. Diclofenac lactam (2) was not observed until 24 h of incubation (30.8 nM), and after 48 h (42.1 nM) its amount constituted 21% of the total initial diclofenac bonded to SYN321. Similarly, linker (3) could be detected after 24 h, with concentrations of 37.2 and 146 nM at 24 and 48 h, respectively.

**Table 2:** SYN321 hydrolysis profile in human plasma or synovial fluid. a) 1 µg mL^−1^ of SYN321. b) Quantification limit 10 nM. c) In parenthesis, amount relates to the initial diclofenac bonded to SYN321 for (1) and (2) and of initial linker for (3). d) For compound 3 the quantification limit in synovia was 100 nM. LLOQ = lower limit of quantification. All the data is the calculated media of two experiments. Values with “‐“ are BLOD (Below Limit of Detection).

Entry	Matrix	Time [h]	1 [nM]^b^	2 [nM]^b^	3 [nM]^b^
1^a^	Plasma	0	<LLOQ	–	–
2^a^	Plasma	4	11.4	–	–
3^a^	Plasma	6	14.0	–	–
4^a^	Plasma	24	85.2	30.8	37.2
5^a^	Plasma	48	125 (67%)^c^	42.1 (21%)^c^	146 (74%)^c^
					
6^a^	Synovia	0	–	–	– ^d^
7^a^	Synovia	4	–	–	– ^d^
8^a^	Synovia	6	–	–	– ^d^
9^a^	Synovia	24	79.0	<LLOQ	– ^d^
10^a^	Synovia	48	129	<LLOQ	– ^d^

When the experiment was conducted in human synovial fluid (Table 2, entries 6–10), the amount of diclofenac (1) could not be detected until 24 h (79.0 nM), and it was similar to the amount released in human plasma at 24 (85.2 versus 79.0 nM) and 48 h (125 versus 129 nM). Diclofenac lactam (2) was detected at the same time points (24 and 48 h), but it could not be quantified as the amount after 24 and 48 h was below the lower limit of quantification (10 nM). Unfortunately, the limit of quantification for compound 3 (linker) was 10 times higher in synovial fluid than in plasma due to the complexity of the matrix sample, making more difficult the comparison of the results. Overall, the release profile of diclofenac from SYN321 exhibited similarities in both plasma and synovial fluid, with comparable concentrations observed in both media. Notably, the formation of diclofenac lactam was reduced within the synovial fluid.

### 2.7 Assessment of SYN321 Diclofenac Release in the Mechanically Active uBeat MultiCompress

**Figure 5:**
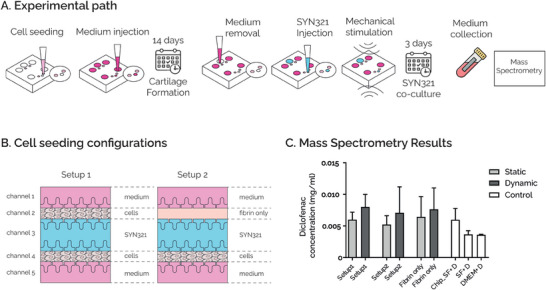
A) Experimental path: hACs embedded in fibrin gel were cultured in the uBeat® MultiCompress Platform for 14 days to achieve mature cartilage constructs. After removing culture medium from therapeutic product‐dedicated channel, SYN321 was injected and co‐cultured with cartilage tissues for 3 days, either in static or dynamic conditions. Culture medium was collected at the end of the culture period for mass spectrometry analysis. B) Cell seeding configurations. In Setup 1 both channels 2 and 4 were injected with cell‐laden hydrogel; in Setup 2 channel 4 was injected with cell‐laden hydrogel and channel 2 was loaded with fibrin only. C) Mass‐Spectrometry Results. Diclofenac concentration in devices cultured with Setup1 and with Setup2, either in static or dynamic conditions. Results are compared with devices loaded with fibrin only in both channels 2 and 4, and with three technical controls, i.e. diclofenac diluted in serum‐free medium and injected in the devices (Chip_SF+D), diclofenac diluted in serum‐free medium without being injected in the devices (SF+D), and diclofenac diluted in DMEM without being injected in the devices (DMEM+D).

Diclofenac release due to hydrolysis of SYN321 bonds was further assessed in the uBeat MultiCompress platform, to dissect the role of a mechanically active environment in the process. Briefly, hACs embedded in fibrin gel were cultured in the device for 14 days in chondrogenic medium (**Figure** **5**A), in two different seeding configurations: i) Setup 1, where both channel 2 and channel 4 were injected with cell‐laden hydrogel (, and ii) Setup 2, where channel 4 was injected with cell‐laden hydrogel while channel 2 was loaded with fibrin only (Figure 5B). After cartilage maturation, SYN321 was successfully injected in the therapeutic product channel (i.e., channel 3) in all the devices, and co‐cultured for 3 days, either in static or dynamic conditions (Figure 5A). Mass Spectrometry analysis performed on cell culture supernatant at the end of the culture period (Figure 5C) detected diclofenac in all the tested conditions, meaning that diclofenac was effectively released from SYN321. In details, measured diclofenac concentration was higher than 4.5 µg mL^−1^. Diclofenac was also detected at similar levels in control devices where both channels 2 and 4 had been loaded with fibrin gel only, indicating that the ester bonds between diclofenac and NaHA were hydrolysed in the uBeat MultiCompress platform independently from the presence of hACs. Notably, diclofenac concentration in devices filled with diclofenac only dissolved in serum‐free medium without any gels (i.e., technical control CHIP_SF+D) was higher than 4.5 µg mL^−1^. Conversely, a diclofenac concentration lower than 4.5 µg mL^−1^ was detected in solutions where the drug was dissolved either in serum‐free medium or in DMEM that had not been injected in the platforms (i.e., technical control SF+D and DMEM+D, respectively). This observation, along with the results of an evaporation rate test within the uBeat MultiCompress platform (detailed in Supporting Information, Figure S3), suggested that the medium could have partially evaporated in conditions evaluated inside the platforms, thus resulting in an increased concentration of released diclofenac in the collected medium. Remarkably, all experimental setups showed higher diclofenac concentrations and evaporation rates under dynamic conditions compared to static conditions.

Overall, these findings indicate that SYN321 can be successfully cultured up to three days within the uBeat MultiCompress platform and that diclofenac is effectively released from SYN321 within the device within this timeframe.

### 2.8 Effect of SYN321 in a Rat MIA Model of OA

**Figure 6:**
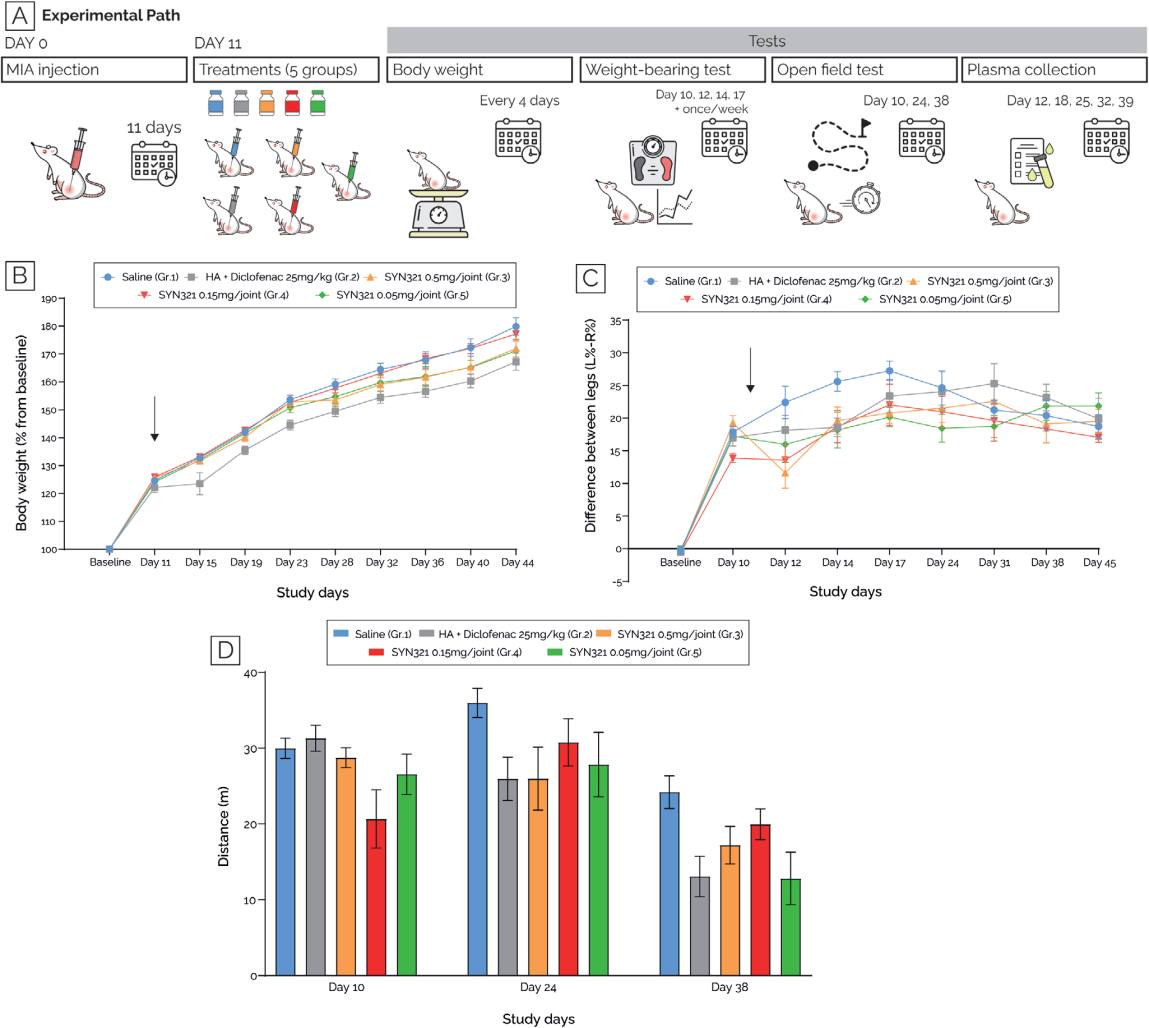
Effect of SYN321 in a rat MIA model of OA. A) A graphical representation of the experimental set‐up is reported. B) Animals’ body weight measured at the beginning of the study (day ‐1, baseline) and every 4 days, starting from day 11. Results are reported as percentage of the baseline. C) Results of the weight bearing tests, showing the weight‐bearing difference between the left (i.e. healthy) and right (i.e. where MIA was administered unilaterally) legs, measured at the beginning of the study (day ‐1, baseline) and on study days 10, 12, 14, 17, and then once weekly. Black arrows in both graphs indicate day 11, i.e. day of treatment administration. D) Results of the open‐field test, showing rats’ walking distance on day 10, 24 and 38. In each of these tests five groups were compared, i.e. receiving (i) saline solution (Group 1, in light blue), (ii) HA and diclofenac (Group 2, in grey), (iii) 0.5 mg per joint SYN321 (Group 3, in orange), (iv) 0.15 mg per joint SYN321 (Group 4, in red), (v) 0.05 mg per joint SYN321 (Group 5, in green).

The SYN321 efficacy on relieving OA symptoms was evaluated in the rat MIA model of OA, utilizing incapacitance (weight bearing) and open‐field tests. The experimental design allowed for the assessment of the effects of different interventions on the knee joint, with Group 1 serving as a control receiving saline, Group 2 receiving HA and diclofenac, and Groups 3–5 receiving SYN321 with potential variations dose levels (0.5, 0.15 and 0.05 mg per joint, **Table** **3**, **Figure** **6**A).

**Table 3:** Treatment groups to evaluate the effectiveness of SYN321 in the rat MIA model. All treatments were administered once on study day 11. ^a^Group size: N = 10. ^b^50 µL per joint. ^c^Animals in group 2 received diclofenac (25 mg kg^−1^) administered orally (PO) once on study day 11.

Group^a^	Treatment	Route	Dose level [mg per joint]^b^
1	Saline	IA	N/A
2	HA + diclofenac^c^	IA + PO	0.5
3	SYN321	IA	0.5
4	SYN321	IA	0.15
5	SYN321	IA	0.05

As shown in Figure 6B (Tables S5 and S6, Supporting Information), all animals experienced weight gain throughout the study. Notably, animals in group 2, i.e., treated with HA + diclofenac, exhibited on average lower weight gain compared to the saline‐treated group (group 1). In contrast, animals treated with SYN321 (groups 3–5) displayed a higher weight gain rate than group 2 (HA + diclofenac) and a similar rate to group 1 (saline) throughout the study. Moreover, as reported in Figure 6C and Table S7 (Supporting Information), animals evenly distributed their body weight on both legs, prior to MIA injection. By study day 10, following unilateral MIA administration to the right knee, animals exhibited a tendency to favor the healthy leg (left), resulting in an increased difference between the two hind legs compared to the baseline level. Upon treatment administration, animals in the vehicle‐treated group (group 1) exhibited an increasing weight‐bearing difference between the two hind legs, reaching the maximum difference in weight‐bearing measurements on testing day 17 (27.23% ± 1.49%, compared to 17.83% ± 0.85% observed on study day 10). After day 17, the difference between the legs began to diminish, reaching a value by day 45 comparable to that observed on study day 10. Treatment with SYN321 at a dose level of 0.5 mg per joint (group 3) led to a statistically significant reduction in the weight‐bearing difference between the two hind legs on testing day 12, compared to the vehicle group (11.60% ± 2.34% versus 22.39% ± 2.48% for the vehicle). On study days 14 and 17, treatment with either dose level of SYN321 showed a trend of reduction in the weight‐bearing difference between the two hind legs compared to the saline‐treated group. However, these reductions were not statistically significant. Throughout the study, treatment with HA in combination with a single oral administration of diclofenac (group 2) did not yield a significant reduction in the weight‐bearing difference between the two hind legs compared to the vehicle group. Finally, open‐field tests were performed on study days 10, 24, and 38 (Figure 6D). Briefly, animals were placed in an open field apparatus for 5 min, and their walking distance was measured. On study day 24, animals treated with saline (group 1) covered the longest distance (35.96±1.93m), with only animals treated with the mid‐dose of SYN321 (0.15 mg per joint; Group 4) approaching that distance: 30.76±3.12m. On study day 38, all animals covered less distance, with those treated with the mid‐dose of SYN321 (0.15 mg per joint; Group 4) covering more distance than all other treatment groups: 19.95±2.02m versus 13.07±2.66m for animals treated with HA + diclofenac (group 2).

Overall, based on the data obtained in this study, animals treated with SYN321 at all doses (groups 3–5) exhibited greater weight gain throughout the study compared to those treated with HA + diclofenac (group 2). Notably, treatment with the high dose level of SYN321 (0.5 mg per joint; group 3) significantly reduced weight‐bearing differences between the intact and injured legs on study day 12 compared to the saline‐treated group. Furthermore, molecular analysis was exploited to measure the level of diclofenac and linker in the rat plasma throughout the study. All data points fell below the lower limit of quantification (LLOQ) of 3 ng/mL (data not shown), supporting a minimal systemic exposure of diclofenac after IA administration of SYN321.

### 2.9 uBeat MultiCompress^®^ Enables to Elucidate the Efficacy of SYN321 to Reduce OA Traits under Hyperphysiological Mechanical Load

Upon evidence of a positive trend of SYN321 in alleviating symptoms in the MIA model, the previously qualified uBeat MultiCompress platform was used to dissect the cellular and molecular mechanisms underlying SYN321 effect.

**Figure 7:**
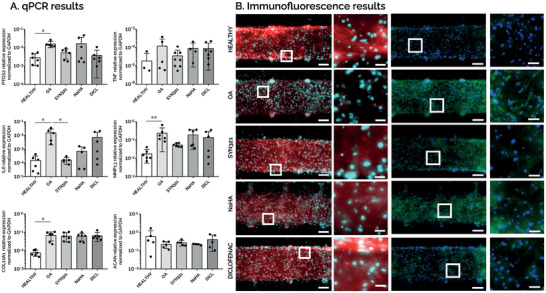
A) qPCR results. Gene expression analysis performed at day 23, comparing static samples (HEALTHY), HPC‐induced pathological samples (OA), OA samples co‐cultured with SYN321 (SYN321), OA samples co‐cultured with NaHA (NaHA), and OA samples supplemented with diclofenac only (DICL). n≥6 (undetected values are not represented). B) Immunofluorescence results. Immunofluorescence stainings showing DAPI (in light blue), aggrecan (in red), and MMP13 (in green) for every condition. Scalebar = 100µm; scalebar magnified pictures = 20 µM.

To this aim, a cartilage model was successfully generated in uBeat MultiCompress platform by culturing hACs for 14 days in static conditions in chondrogenic medium. Subsequently, osteoarthritis traits were recapitulated in the model by applying one week of HPC (i.e., 30%) as previously described. The effect of SYN321 was then evaluated after three days of treatment in dynamic conditions and compared to positive controls treated either with NaHA or diclofenac only (dissolved in culture medium at 4.5 µg mL^−1^). Gene expression analyses are reported in **Figure** **7**B, where each gene expression level was normalized to *GAPDH* expression. SYN321 treatment resulted in an anti‐inflammatory effect in the model, inducing a decrease in the expression of *TNF*, *PTGS2*, and *IL6*, as compared to non‐treated OA control. Notably, the expression of *IL6* in SYN321‐treated samples was significantly reduced as compared to OA samples (*P<0.05) and it was comparable with healthy condition expression level. The downregulation of these pro‐inflammatory genes was less marked in the positive controls. In particular, treatment with diclofenac decreased only *PTGS2* expression, while the treatment with NaHA decreased only *IL6* expression. Additionally, the variability of NaHA and diclofenac data was higher as compared to SYN321.

Also, SYN321 played a role in reducing matrix degradation. *MMP13* expression significantly increased in the OA condition (** P<0.01) with respect to healthy controls. Treatment with SYN321 resulted in a decreased *MMP13* expression with respect to OA control. On the contrary, NaHA‐ and Diclofenac‐treated samples showed an *MMP13* gene expression comparable to OA control.

Regarding OA hypertrophic traits, no treatment condition demonstrated a reduction of matrix calcification. *COL10A1* expression indeed significantly increased in the OA condition (* P<0.05) with respect to the healthy control, while no variation was detected in any treatment conditions (i.e., SYN321, NaHA, Diclofenac) with respect to OA samples. Concerning the chondrogenesis indicator, at the gene level no treatment conditions (i.e., SYN321, NaHA and diclofenac) restored the *ACAN* expression at similar levels as compared to healthy control.

Immunofluorescence results are shown in Figure 7B. Regarding matrix deposition, healthy control showed a matrix rich in aggrecan, while the same protein was almost absent in the OA condition. Treatments resulted in matrices richer in aggrecan as compared to OA condition, especially in the case of SYN321 and diclofenac. From a matrix degradation point of view, MMP13 was barely expressed in the healthy control, while OA condition caused a marked increase in MMP13‐positive cells. SYN321 treatment caused a decreased MMP13 expression as compared to OA samples, confirming the results obtained at the gene level. Diclofenac‐treated samples also showed a reduced MMP13 expression at protein level, while in NaHA condition MMP13 expression was comparable with OA condition.

Overall, these results confirmed that uBeat MultiCompress platform was suitable to test the efficacy of injectable therapeutic formulations (i.e., SYN321) from a molecular and cellular perspective. SYN321 was proven to exhibit an anti‐inflammatory effect and to play a role in reducing matrix degradation both at gene and protein level.

## 3 Discussion

OA is a prevalent degenerative joint disorder characterized by articular cartilage breakdown and synovial joint failure, with a significant impact on patient quality of life.^[^ ^32^ ^]^ Current therapeutic strategies for OA are limited and often fail to address the underlying pathophysiology of the disease.^[^ ^33^ ^]^ Treatment strategies typically include analgesics, glucocorticoids, and NSAIDs such as ibuprofen, celecoxib, and diclofenac, to alleviate pain and inflammation.^[^ ^7^, ^8^, ^9^ ^]^ However, despite their proved therapeutic potential, systemic side effects associated with long‐term NSAID use are a significant concern, prompting interest in targeted IA therapies, that in turn present challenges such as rapid clearance from synovial fluid.^[^ ^10^, ^11^, ^12^, ^13^ ^]^ Innovations in polymer‐drug conjugates, particularly the combination of HA viscoelastic hydrogels with NSAIDs like diclofenac, show promise for sustained NSAID release in the joint.^[^ ^13^, ^14^, ^15^, ^16^ ^]^ The successful development of such injectable NSAIDs formulation is nevertheless hampered by a lack in preclinical OA models providing a faithful recapitulation of the human pathophysiology, while being permissive to the screening of injectable and viscous therapeutic products.

The here presented uBeat MultiCompress platform represents a novel approach in microscale cell culture technologies, specifically designed for the study of anti‐OA injectable therapeutic treatments. The device's architecture is an evolution from a previous platform (named uBeat) able to deliver uniform and confined mechanical compression to 3D cellular structures and specifically tailored for exerting HPC on cartilage micro‐tissues to induce OA traits.^[^ ^28^ ^]^ The requirement for applying mechanical stimulation to cell cultures, particularly in the context of cartilage tissue engineering, is well recognized in scientific literature. Mechanical forces are known to play a crucial role in the maintenance and development of cartilage, as well as in the initiation of pathological conditions. Studies have shown that hACs respond to mechanical stresses by altering their metabolism, which can influence tissue growth and degradation.^[^ ^34^, ^35^, ^36^ ^]^ Moreover, alterations in the mechanical microenvironment of the joint, e.g. due to misalignment, abnormal joint shape, obesity and microfractures, have been established as pivotal clinical determinants in the development of OA. In this context, our group previously illustrated the ability to model joint biomechanics in vitro using the uBeat platform, demonstrating how a precise control over mechanical forces obtained within this system can recapitulate such a mechanically driven OA onset, an aspect often lacking in traditional in vitro cartilage models. As an evolution of our previous platform, the here presented uBeat MultiCompress platform was designed with the aim to apply the same precise control over the mechanical environment not only to the cartilage microtissues, but also to injectable formulations hosted in close contact to them. The possibility to host injectable therapeutic feature is crucial, as many anti‐OA therapeutic products, like HA‐based solutions, have medium‐to‐high viscosity,^[^ ^37^ ^]^ posing challenges in their integration within microfluidic environments.

The technical characterization of the platform, including mechanical and compatibility assessments with viscous therapeutic products, demonstrated the device efficacy in maintaining uniform compression and accommodating various therapeutic formulations without leakage or structural compromise. Our observations suggested a slight position‐dependent distribution for longitudinal strains but not for lateral strains, being still all of them negligible when compared to the predominant 30% compression level applied in the z‐direction. These data demonstrate the ability of the uBeat MultiCompress platform to incorporate injectable therapeutic formulations into its design, thus presenting a novel avenue for testing potential treatments for OA.

To demonstrate the platform's versatility and potential in OA treatment research, a biological qualification was first performed using commercially available therapeutic products like Supartz and the platform was subsequently used to explore a novel anti‐OA formulation (i.e., SYN321). In details, cartilage microtissues were generated through the 3D culture of hACs embedded in fibrin gel, that were allowed to mature inside the platform for 14 days. The observed upregulation of aggrecan gene expression and its high deposition in the ECM was in good accordance with the results obtained by Occhetta et al.^[^ ^28^ ^]^ and indicated healthy cartilage tissue formation, as aggrecan is a constitutive component of the cartilage ECM and its presence is indicative of a healthy chondrocyte activity.^[^ ^38^ ^]^ Moreover, the upregulation of *PRG4*, a gene that encodes for lubricin which is crucial for joint lubrication, suggested proper tissue maturation.^[^ ^39^ ^]^ Of note, a supraphysiological level of collagen type‐I was detected in our culture system. Although the chondrocytes were of primary origin, they were expanded in 2D with factors that promote de‐differentiation into fibroblast‐like cells.^[^ ^40^ ^]^ This acquired phenotype was partially reversed during the 3D culture in the CoC device with appropriate chemical conditioning. After cartilage maturation, the successful induction of an OA phenotype through mechanical overload was evidenced by the upregulation of inflammatory genes such as *PTGS2*, encoding for COX‐2, and *IL6*, as well as the increased expression of degrading mediators such as *MMP13*. Moreover, the increase in *COL10A1* expression, pointing towards chondrocyte hypertrophy, as well as the decreased presence of aggrecan at the protein level, indicating loss of cartilage matrix components, reflected the pathological changes observed in OA joints.^[^ ^41^ ^]^ Finally, the investigation into mechanoreceptor activation revealed intriguing findings. Chondrocytes have been demonstrated to sense mechanical loads mainly through PIEZO1, PIEZO2 and transient receptor potential vanilloid TRPV4, mechanosensitive ion channels highly expressed in numerous tissues throughout the body. These mechanosensitive Ca2+‐permeating channels are robustly expressed in primary articular chondrocytes and trigger force‐dependent cartilage remodeling and injury responses. PIEZO channels specifically have been implicated in pathogenic remodeling of cartilage in response to hyperphysiological loading.^[^ ^42^ ^]^ In our system, both *PIEZO2* and *TRPV4* genes resulted upregulated upon 7 days of mechanical overload, thus suggesting a direct effect of confined compression on activation of such mechanoreceptors in accordance with literature data.^[^ ^43^ ^]^ *PIEZO1* expression was conversely not modulated after 7 days of HPC in our system, in discrepancy with previous observation of *PIEZO1* protein increase in response cyclic loading to human chondrocytes encapsulated in PEG hydrogels.^[^ ^44^ ^]^ This may be explained due to a possible mismatch in the observation temporal window, being *PIEZO1* channel directly and rapidly activated by mechanical cues (τ_ac__Piezo1 < 5 msec) with rapid subsequent inactivation time (τ_inac__Piezo1≈ 16 msec).^[^ ^45^ ^]^

The model recapitulated inside the uBeat MultiCompress platform was thus exploited to test the efficacy of Supartz, a clinically approved HA‐based therapeutic. Specifically, Supartz is a viscoelastic solution composed of highly purified HA (average molecular weight 900 kDa) and was the first HA product globally to receive clinical approval for treating knee OA pain through IA application.^[^ ^29^ ^]^ In our model, Supartz was proven to induce down‐regulation of *COX‐2*, as previously reported in an in vitro study on human chondrosarcoma SW‐1353 cells stimulated with IL‐1β, and resembling properties of primary chondrocytes from OA subjects.^[^ ^46^ ^]^ Also, this result was in good accordance with the preclinical study of Asari et al.,^[^ ^47^ ^]^ where in a canine model of knee OA induced by anterior cruciate ligament transection, IA administration of Supartz was found to decrease the concentration of prostaglandin E2 (PGE2), i.e., an inflammatory factor product of the *COX‐2* enzyme, in the synovial fluid. Moreover, the observed reduction in *IL6* expression in Supartz‐treated samples is a critical finding, as IL6 is widely recognized for its role in the pathophysiology of OA, contributing to inflammation and joint destruction.^[^ ^48^ ^]^ However, differently from what obtained from Furuta et al. on human chondrocytes from OA patients,^[^ ^49^ ^]^ Supartz did not cause a reduction in *MMP13* expression of OA cartilage micro‐tissues cultured in the uBeat MultiCompress platform. Finally, no noticeable alterations in *ACAN* gene expression were observed in samples treated with Supartz. As a comparison, in the mouse transtibial resection model for knee OA presented from Li et al., IA injections of Supartz FX were shown to enhance the expression of type‐II collagen and aggrecan genes in the cartilage.^[^ ^50^ ^]^ In conclusion, the lack of significant changes in *MMP13* and *ACAN* expression in Supartz‐treated samples in the uBeat MultiCompress Platform may indicate that the primary action of Supartz in this model is modulation of inflammation rather than direct inhibition of matrix degradation and promotion of matrix synthesis. Overall, these findings proved that the MultiCompress platform provides a novel and effective way to evaluate the therapeutic potential of OA treatments, with the therapeutic formulation being injected in the platform, cultured in direct contact with 3D OA cartilage microtissues and mechanically stimulated together with them, resembling the in vivo environment.

While numerous studies have highlighted the advantages of HA‐based therapies, such as decreased pain levels and enhanced joint lubrication, the use of IA HA for knee OA has not been firmly established. Due to the variable efficacy among patients and the lack of consistent evidence supporting its benefits, most guidelines generally do not endorse IA HA as a primary treatment modality for OA.^[^ ^15^, ^51^ ^]^ Thus, as previously introduced, researchers’ focus is on combining HA injections with NSAIDs, that is crucial for several reasons. First, NSAIDs can help manage the inflammatory component of OA, providing additional pain relief and reducing joint swelling. Second, they can enhance the overall effectiveness of treatment by targeting different pathways involved in the disease process. Finally, NSAIDs can help extend the duration of symptom relief beyond the immediate effects of HA injections.^[^ ^7^, ^52^, ^53^, ^54^ ^]^ For instance, SI‐613, developed by Seikagaku, employs a proprietary pH‐dependent drug‐binding technique to combine diclofenac with NaHA, leveraging diclofenac's anti‐inflammatory properties and NaHA's lubricating effects. SI‐613 is engineered to ensure sustained release of diclofenac and has demonstrated analgesic and anti‐inflammatory effects in a rat model of silver nitrate‐induced arthritis.^[^ ^55^ ^]^ However, in SI‐613, the linker is connected to the carboxylate of the hyaluronan backbone via an amide bond, hindering linker release and resulting in the formation of metabolites derived from hyaluronan oligosaccharides connected to the linker. Similarly, SYN321, developed by Synartro AB, is a conjugate of diclofenac and modified NaHA, designed to achieve controlled drug release within the joint space and to provide effective and sustained pain relief through the combined actions of diclofenac and NaHA. This synergy is achieved through a novel technology called Hydro‐link, that enables localized injection and gradual release of the active compound in SYN321, prolonging active substance presence over tenfold compared to free diclofenac injections. Moreover, localized injection of SYN321, coupled with gradual release post‐hydrolysis of esters, allows administration of an exceptionally low NSAID dose, mitigating adverse effects associated with systemic diclofenac administration. Previous findings in horses suggest that the equivalent of 6 mg of diclofenac in SYN321 injectable solution could yield robust and sustained pain relief, in contrast to the standard oral regimen of 100–150 mg daily over 14 days.^[^ ^13^ ^]^ Specifically, the hyaluronan backbone in SYN321 is functionalized with succinic acid sodium salt moieties, transforming it into a highly charged polymer to enhance solubility in water, facilitated by the negatively charged side‐structures of succinyl hyaluronan. This mirrors the hydrophilic characteristics observed in the proteoglycan/HA complex within synovial fluid, promoting high hydration.^[^ ^56^ ^]^ Subsequent functionalization of succinyl esters with a lipophilic molecule (i.e., diclofenac ester) allows easy connection of the linker through ester bonds to both hyaluronan and diclofenac. Compared to SI‐613, SYN321 structure is based on ester bonds, which are weaker than amide bonds, facilitating linker hydrolysis from hyaluronan and resulting in endogenous hyaluronan degradation. The preliminary interventional study was conducted on horses by Rhodin et al., to assess potential clinical side effects of SYN321 and gather preliminary data on the concentration and duration of diclofenac in the synovial fluid.^[^ ^13^ ^]^ According to the results of the study, side effects were minimal, with only mild signs of synovitis, local irritation, joint effusion, swelling, and occasional subtle lameness observed. Moreover, the study found that SYN321 provided prolonged exposure of diclofenac in horses' synovial fluid, as evidenced by consistent concentration levels. However, the correlation between these findings and clinical efficacy remained uncertain, suggesting a need for further preclinical research to explore SYN321's potential for treating OA.

Here, hydrolysis profile and release kinetics of SYN321 were first assessed in both human plasma and synovial fluid. Of note, the employed experimental set‐up imposes constraints on the free movement of molecules and differs from the dynamic environment of the synovial cavity, where molecules can freely diffuse within the synovial fluid and traverse the synovial membrane, thus facilitating metabolite dispersion. To address this limitation, concentrations of SYN321 were reduced by four orders of magnitude compared to those typically administered in clinical settings (i.e., assuming 98 mg of SYN321 in a 7 mL synovia volume^[^ ^57^, ^58^ ^]^). Three primary metabolites were identified, including diclofenac, diclofenac lactam, and the linker compound. Interestingly, the release profile of diclofenac exhibited similarities between human plasma and synovial fluid, with comparable concentrations observed over the 48‐hour period. However, the formation of diclofenac lactam and linker was notably reduced in synovial fluid compared to plasma, suggesting differential metabolic processes within the joint environment. These findings align with previous research indicating variations in drug metabolism between systemic circulation and synovial compartments, likely influenced by factors such as pH, enzymatic activity, and matrix composition.^[^ ^59^ ^]^ Considering that the nature of the matrix and the concentration of SYN321 and its released compounds likely impact hydrolysis kinetics, elucidating a clear pathway for diclofenac release in SYN321 is challenging due to numerous influencing factors, and accurately simulating the synovial cavity poses significant difficulty. One hypothesis is that the hydrolysis of the ester bond between the linker and hyaluronan is primarily non‐enzymatic, potentially due to steric hindrance and charge effects, influenced by the media pH. Additionally, the hydrolysis of the bond between linker and diclofenac may be non‐enzymatic when the linker is still connected to the hyaluronan backbone, but predominantly enzymatic when the linker is freed from hyaluronan but connected to diclofenac (i.e., forming a small molecule susceptible to esterase activity).

The release of diclofenac from SYN321 was additionally investigated exploiting the uBeat MultiCompress platform, to assess diclofenac release dynamics within a physiologically relevant environment, incorporating hACs and mechanical stimulation. The presence of hACs within the uBeat MultiCompress platform did not significantly alter diclofenac release kinetics from SYN321. Mass spectrometry analysis revealed comparable diclofenac concentrations in devices with chondrocytes and in those without, suggesting that chondrocytes did not markedly influence the hydrolysis of ester bonds within SYN321. Conversely, diclofenac release was enhanced under dynamic conditions compared to static conditions. While this result is partially related to a higher evaporation rate measured in dynamic condition, it may also suggest a potential role of mechanical stimulation in modulating drug release kinetics. In addition to increasing evaporation, mechanical stimulation may indeed facilitate the disruption of ester bonds within SYN321, leading to increased diclofenac release.

Then, the efficacy of SYN321 in alleviating OA symptoms was comprehensively evaluated in a rat MIA model. The study design facilitated the comparison of different intervention strategies, including SYN321 at different concentrations, HA combined with orally administered diclofenac, and saline control. While animals treated with HA and diclofenac showed lower weight gain than the saline‐treated group, animals treated with SYN321 displayed a higher weight gain rate, approaching the rate observed in the saline group. This observation suggests that SYN321 may have a less mitigating impact on weight gain compared to the HA and diclofenac combination. Conversely, the combined treatment of HA with diclofenac did not result in a significant reduction in weight‐bearing differences between intact and injured legs, while SYN321 treatment, particularly at a higher dose (0.5 mg per joint), resulted in a significant reduction compared to the saline‐treated group. This suggests a potential therapeutic benefit of SYN321 in ameliorating OA‐induced asymmetry in weight distribution, as compared to HA and diclofenac. Furthermore, open‐field test results further support the superior efficacy of SYN321 compared to HA and diclofenac. Animals treated with SYN321, particularly at the mid‐dose (0.15 mg per joint), covered a greater distance in the open‐field test compared to those receiving HA and diclofenac. This indicates improved locomotor function and mobility with SYN321 treatment, aligning with the observed reductions in weight‐bearing differences. Notably, the mean ratio between the two hind legs in animals treated with SYN321 remained lower than that of animals treated with HA and diclofenac throughout the study, despite the SYN321 group receiving doses 20–200 times lower than the diclofenac dose received orally. In fact, IA administration allows diclofenac to directly target the affected joint, bypassing systemic circulation and achieving higher concentrations at the site of action. This localized delivery enhances the therapeutic efficacy of diclofenac, leading to comparable or even superior outcomes with lower doses. In contrast, oral administration of diclofenac results in systemic distribution, leading to lower concentrations reaching the joint and potentially reduced effectiveness in alleviating OA symptoms. Therefore, the observed results underscore the advantage of IA administration in optimizing the therapeutic effect of diclofenac for OA management. Finally, absence of diclofenac and linker in rat plasma throughout the study indicated minimal systemic exposure of diclofenac following IA administration of SYN321. This finding is consistent with the expected minimal systemic exposure of diclofenac after IA administration of SYN321 and highlights the potential reduction in the occurrence or severity of side effects associated with oral NSAIDs administration.

Despite the significant findings observed in the rat MIA model, no indications could be obtained about the cellular and molecular mechanisms underlying the beneficial effect of SYN321. Thus, we exploited the uBeat MultiCompress platform to evaluate SYN321 mode of action at gene and protein level, in a physiologically relevant human model of OA. In evaluating SYN321 efficacy, the decrease in pro‐inflammatory gene expression (*TNFα, IL6*) and reduction in matrix degradation markers (*MMP13*) were noteworthy. The findings on *MMP13* expression under SYN321 treatment, that were confirmed at protein level, are crucial as MMP13 is a well‐known mediator of cartilage breakdown in OA.^[^ ^60^ ^]^ Moreover, as compared to OA untreated samples, SYN321‐treated samples exhibited downregulation of *PTGS2*, encoding for COX‐2, i.e., a key enzyme involved in the inflammatory process that is often targeted by OA therapies.^[^ ^61^ ^]^ Interestingly, the study revealed that SYN321 did not significantly affect the expression of *ACAN* at gene level, suggesting that its primary mechanism may be anti‐inflammatory rather than anabolic. However, at protein level, treatment with SYN321 resulted in matrices with higher aggrecan levels compared to untreated samples. Finally, SYN321 treatment was not proven to have any effect on *COL10A1*, thus suggesting no effect on reverting the hypertrophic phenotype characteristic of OA chondrocytes.^[^ ^62^ ^]^ Generally, SYN321 showed a better therapeutic profile as compared with control treatments, including NaHA and diclofenac. Overall, the results of this case study suggested that SYN321 effectively i) reduced the inflammatory response observed in the OA model, ii) decelerated the initiation of matrix‐degrading processes at the intracellular level, iii) moderately reinstated aggrecan expression in the ECM at the protein level, yet iv) did not demonstrate any impact on diminishing cartilage calcification. Thus, this case study positions SYN321 as a promising therapeutic candidate for OA and further validates the uBeat MultiCompress platform's utility in OA research, bridging the gap between traditional in vitro studies and potential clinical applications.

Future advancements of this work will include conducting long‐term studies using the uBeat MultiCompress platform to monitor the long‐term effects of therapeutic agents like Supartz and SYN321, aiming at understanding the sustained efficacy of treatments, as well as exploring a broader range of therapeutic agents to expand the platform's applicability in screening novel therapeutics. Adapting the platform for higher throughput screening of multiple drug candidates simultaneously could significantly accelerate the pace of drug discovery in OA.

Moreover, the current OoC model effectively simulates the cartilage microenvironment and the mechanical stresses associated with OA (main target of SYN321). However, it is well‐recognized that the immune microenvironment, particularly the presence and activity of macrophages and other immune cells, plays a crucial role in the pathophysiology of OA.^[^ ^63^ ^]^ Inflammatory responses in cartilage are significantly influenced by the interaction between chondrocytes and immune cells, with macrophages being key players in mediating inflammation and tissue degradation through the secretion of pro‐inflammatory cytokines and matrix‐degrading enzymes.^[^ ^64^ ^]^ The absence of an immune compartment in our current model may limit its ability to fully recapitulate the complex inflammatory processes occurring in OA joints. Future development will be focused on incorporation of additional joint‐related and immunocompetent compartments (e.g., a synovium compartment, that may include resident macrophages and synovial fibroblasts, as well as circulating immune cells).

Finally, the versatility of the mechanical setup and the layout of the uBeat MultiCompress platform present opportunities for its application beyond OA. The platform's ability to deliver uniform mechanical compression to 3D cellular structures can be leveraged to model various musculoskeletal disorders, such as rheumatoid arthritis, tendinopathy, and intervertebral disc degeneration, where mechanical stress plays a critical role in disease progression.^[^ ^65^, ^66^ ^]^ Furthermore, the platform can be adapted to simulate different pathological mechanical environments, allowing researchers to study conditions such as mechano‐transduction in cancer metastasis, fibrosis, and cardiovascular diseases where tissue stiffness and mechanical forces are pivotal.^[^ ^67^, ^68^, ^69^, ^70^, ^71^ ^]^ By incorporating different cell types and extracellular matrix compositions, and modifying the mechanical stimulation protocols, the platform could be customized to recreate the specific biomechanical and biochemical conditions of a wide range of tissues and diseases.

## 4 Conclusions

The uBeat MultiCompress platform emerges as a novel tool tailored for studying anti‐OA injectable therapeutics. Its ability to replicate mechanical compression on cartilage microtissues while accommodating viscous formulations addresses critical gaps in traditional in vitro models. The platform's efficacy and versatility were qualified through mechanical and compatibility assessments with therapeutic products like Supartz and SYN321. In particular, SYN321, a novel drug candidate based on modified NaHA bounded to a diclofenac derivative, showed promising results in mitigating OA symptoms in vivo and demonstrated to have a beneficial effect in reducing OA traits in vitro. Studies inside the uBeat MultiCompress platform elucidated SYN321 mechanisms of action, highlighting its anti‐inflammatory effects and potential for mitigating matrix degradation.

Overall, the uBeat MultiCompress ability to replicate the complex mechanical environment of the human joint and test therapeutic formulations directly within a 3D cellular matrix offers a promising tool for OA research and drug development. Future studies could leverage this technology to explore new therapeutic pathways and deepen our understanding of joint pathophysiology.

## 5 Experimental Section

### Design and Fabrication of the Microfluidic Chip

The layout of the CCL and MAL layers was designed using a computer‐aided design (CAD) software (AutoCAD, Autodesk Ink). In details, the CCL consists of five channels divided by four rows of T‐shaped overhanging posts whose branches are 300 µm wide x 100 µm thick, with each post being spaced by 30 µm from the next. The medium channels (i.e., channels 1 and 5) are 450 µm wide, the cell‐laden hydrogel channels (i.e., channels 2 and 4) are 300 µm wide, while the therapeutic product channel (i.e., channel 3) is 700 µm wide. Channel 3 features one inlet on one side, for product injection, and one reservoir on the other, while channels 2 and 4 feature inlets at both sides, and channels 1–5 feature reservoirs at both extremities. The MAL is composed of three rectangular chambers (3.3 mm x 6.1 mm) connected by a 300 µm wide channel that ends in a 1.5 mm‐diameter inlet (i.e., actuation port). Each chamber is featured with six rows of 30 µm‐diameter circular pillars. Each pillar is 150 µm far from the next ones, while each pillar row is spaced 550 µm from the others.

Master molds for the CCL and MAL layers were then produced in a cleanroom environment (Polifab, Politecnico di Milano) using conventional multi‐layer photolithography technique. SU8 photoresist (MicroChem, USA) was spin‐coated (Karl Süss RC8, Süss Microtec, Germany) on 4″ silicon wafers and used to pattern each layer following a previously developed protocol.^[^ ^72^ ^]^ The Cross‐sectional size of the layers’ features was 2.5 mm (width) x 150 µm (height) for the CCL and 3.3 mm (width) x 50 µm (height) for the MAL. After laser light exposure through a maskless aligner (Heidelberg MLA100, Heidelberg Instruments), wafers with features in relief were cured and developed according to the manufacturer's specification. The microstructured silicon master molds (both CCL and MAL) were then used for the soft lithographic process. First, the master molds were subjected to a silanization treatment. Briefly, the mold surface was exposed to tri‐methylchloro‐ silane vapours (Sigma‐Aldrich) for 30 min at room temperature, in order to prevent the PDMS from sticking to the wafer and help its removal.

PDMS layers (Sylgard 184, Dow Corning; mixing ratio of 10:1 elastome base:curing agent) were thus fabricated through replica‐molding of the master molds. After curing (at 65 °C for 3 h), CCL and MAL PDMS layers were peeled off the mold and assembled after air plasma treatment (i.e., 30 W for 50 s at 0.420 torr) (Harrick Plasma Inc). Wells for medium reservoirs and inlets for hydrogel/therapeutic product injection in the CCL were obtained using biopsy punchers of 4 mm and 1 mm of diameter, respectively. The MAL access port was made with a 1.5 mm puncher to fit a Tygon tube (i.d. 0.50 mm, o.d. 1.5 mm) that connects the device to a compressed air source. The assembled CCL and MAL were finally plasma bonded to a 150 µm‐thick glass coverslip (24×60 mm, #1,5 Menzel Gläser) using the same protocol. Once the whole device was assembled, each actuation chamber resulted separated from the culture unit by 800 µm‐thick PDMS membrane, able to transfer a mechanical stimulation to both cartilage microtissues and to the therapeutic product, when pressure was applied to the actuation chambers.

### Quantification of Mechanical Deformation along x‐ and y‐Axes

The strain field characterizing the micro‐constructs inside the device was experimentally assessed for all the stimulated channels (i.e., 2, 3, and 4) in the lateral and longitudinal directions. Polystyrene Microbeads (10 µm diameter, Sigma Aldrich, USA) were embedded in a fibrin hydrogel, obtained by mixing fibrinogen (FB, Sigma Aldrich) and thrombin (TH, Baxter TISSEEL) to obtain final concentrations of 10 mg mL^−1^ and 2 U mL^−1^, respectively. The microbeads‐laden hydrogel was injected in channels 2,3 and 4 of each chamber and allowed for crosslinking at 37 °C, 5% CO_2_ for 8 min. After cross‐linking, medium channels were filled with PBS. The device was then connected to a compressed air source and images were acquired through an optical microscope in rest condition (i.e., no pressure applied) and in compression condition (i.e., 0.5 bar applied). The minimum pressure to be applied to the actuation compartment to allow the contact between the hanging posts and the flexible membrane was determined as previously described^[^ ^30^ ^]^ and fixed to 0.4 bar.

The mutual distance between 9 couple of beads for each channel was measured along x (i.e., channel width) and y (i.e., channel axial length) direction in rest and compression conditions. Additionally, we computed transversal and longitudinal strains in three different regions along the device's longitudinal axis (i.e., ROI A, B, and C) to examine the position‐dependent strain distribution (Figure 2A).

Strain along x and y was calculated as defined in the equation:

(1) $$\varepsilon \;\varepsilon_{ii}=\frac{\Delta i-\Delta i_{0}}{\Delta i_{0}}$$

where the index *i* indicates the direction (i.e., x or y), and ∆*i* and ∆*i*_0_ correspond to the measured distances after compression and at rest, respectively. According to this formula, a positive strain indicates stretching of the structure, while a negative strain indicates compression. ImageJ software was used to post‐process the acquired pictures and measure microbeads’ distances. Mean value and standard deviation over the couple of beads were calculated in Microsoft Excel, and GraphPAD Prism was used to plot the results.

### Gel Confinement During Injection

In order to assess the feasibility to inject viscous therapeutic products into the dedicated central channel without causing undesired leakages, the presence of cartilage micro‐tissues in channels 2 and 4 was simulated using a microbeads‐laden fibrin solution as described above. After gel cross‐linking, culture medium, i.e., DMEM High Glucose supplemented with 1% Sodium Pyruvate (Gibco), 1% HEPES Buffer Solution 1 M (Euroclone), 1% PSG (Euroclone) and 2% Fetal Bovine Serum (Euroclone), was injected in channel 1, 3, and 5. All the chambers were checked under the microscope to attest fibrin gel cross‐linking (i.e., by verifying stable microbeads confinement inside channels 2 and 4) and subsequently incubated at 37 °C for one hour. Upon removal of medium from channel 3 by aspirating both from its reservoir and the corresponding inlet, different therapeutic products were injected and tested. The injection of three substances was tested, i.e., a commercial one (Supartz), an investigational drug developed by Synartro AB (SYN321) and NaHA 15 mg mL^−1^, purchased from HTL Biotechnology with an intrinsic viscosity of 1.54 m^3^ kg^−1^. Proper gel injection was assessed under the microscope immediately after the procedure. The integrity of the microbeads‐laden fibrin constructs was assessed by ascertaining the absence of microbeads in medium channels (i.e., index of fibrin constructs disruption). In case of presence of microbeads in the medium channel, the injection was considered not successful. Failure rate was calculated as:

(2) $$failure\;rate=\frac{failed\;injections}{total\;n.\;of\;injections}$$

An injection failure rate of 20% was chosen as a threshold above which the therapeutic product inoculation was determined as not feasible. Since the geometry of the device is fixed, the failure rate depends on the product only, so every new product needs to be tested for injectability before proceeding with biological experiments.

### Chondrocytes Expansion

Human primary healthy articular chondrocytes (hACs) (male, 30 y.o., donor 31343, lot. 0000604841, Lonza) were thawed at passage 4 using complete culture medium (i.e., DMEM High Glucose supplemented with 1 mM sodium pyruvate (Gibco), 10 mM HEPES Buffer solution (Euroclone), 100 U mL^−1^ penicillin, 100 µg mL^−1^ streptomycin, 0.292 mg mL^−1^ L‐glutamine (Gibco), 10% Fetal Bovine Serum). Cells were counted using Trypan Blue (Sigma), plated in culture flasks at a density of 5000–5500 cells cm^−2^ and cultured in complete medium supplemented with 1 ng mL^−1^ of transforming growth factor‐β1 (TGF‐β1) and 5 ng mL^−1^ of fibroblasts growth factor‐2 (FGF‐2) in a humidified incubator (5% CO_2_, 37 °C). When reaching approximately 80% of confluence, cells were rinsed with phosphate buffered saline (PBS), harvested using 0.05% Trypsin – EDTA 0.02% (Euroclone), collected in completed medium and used for generating cartilage model within the devices.

### Generation of a Healthy Cartilage Model Inside the uBeat^®^ MultiCompress Platform

A healthy human cartilage model was generated by embedding hACs in a fibrin gel. After harvesting the hACs, aliquots of 1.5 × 10^6^ cells were prepared, suspended in a TH solution in DMEM only, and subsequently diluted 1:1 withFB solution in PBS to obtain a final fibrin concentration of 2 U mL of TH, 10 mg mL^−1^ of FB and a cell seeding density of 50 × 10^6^ cells mL^−1^. The cell laden hydrogel was injected in the dedicated channels (i.e., channels 2 and 4) of the device and cross‐linked in a humidified incubator (5% CO_2_, 37 °C) for 8 min. The medium and the central channels (i.e., channels 1, 5, and 3) and corresponding reservoirs were then filled with chondrogenic medium, namely DMEM containing 2% Fetal Bovine Serum, 10 mM Hepes, 1 mM sodium pyruvate, 100 U mL^−1^ penicillin, 100 µg mL^−1^ streptomycin, 0.292 mg mL^−1^ L‐glutamine and supplemented with Insulin 10 µg mL^−1^, 0.1 mM ascorbic acid 2‐phosphate, 10 ng mL^−1^ Transforming growth factor beta‐3 (TGF‐β3) and aminocaproic acid (ACA) 2 mg mL^−1^. Cells were cultured for 14 days and medium was changed every other day decreasing the concentration of ACA to 1.6 mg mL^−1^ at day 2 and 1.2 mg mL^−1^ at day 4 and following.

### Induction of an OA Phenotype through Mechanical Overload

After 14 days of cartilage maturation, the devices were connected to uBox (BiomimX Srl) and mechanically actuated by applying a HPC of 30% at 1 Hz for 7 days. In particular, the “uKnee pattern” resembling the daily walk was chosen by applying 2 h of stimulation, 4 h of rest, 2 h of stimulation, and 16 h of rest.^[^ ^28^ ^]^ Control devices were cultured under static conditions. Chondrogenic medium was changed every other day. After 7 days of HPC the samples were collected for reverse transcription quantitative real‐time PCR (RT‐qPCR) and immunofluorescence stainings as described below.

### Qualification of the Platform with Supartz®

After 14 days of cartilage maturation and 7 days of HPC to induce OA traits, culture medium was removed from all the reservoirs and channels, including the therapeutic product‐dedicated channel (i.e., channel 3) in each platform. Supartz was transferred into a 0.5 mL Eppendorf from the syringe and then injected in the channel 3 of each chamber and in the corresponding reservoir. The remaining medium channels and reservoirs were filled with serum free medium, namely DMEM containing 10 mM Hepes, 1 mM sodium pyruvate, 100 U mL^−1^ penicillin, 100 µg mL^−1^ streptomycin, 0.292 mg mL^−1^ L‐glutamine and supplemented with 0.1 mM Ascorbic Acid 2‐phosphate. After three days of treatment under HPC, the samples were collected for RT‐qPCR and immunofluorescence as described below. Devices where Supartz was not injected were used as controls (i.e., “OA controls”).

### SYN321 – Synthesis

SYN321 was synthesized in a convergent 4 step synthesis from the starting materials NaHA, succinyl anhydride, diclofenac, and *N*‐Boc‐protected 2‐(2‐aminoethoxy)ethanol following the reported procedure (Figure S2, Supporting Information).^[^ ^13^ ^]^ The detailed protocol is reported in Supporting Information. Briefly, NaHA from bacterial fermentation (A) was reacted with succinyl anhydride (B) to form the ester intermediate C (step 1). Two succinyl groups per hyaluronan disaccharide unit were added. Diclofenac (1) was reacted with *N*‐Boc‐2‐(2‐aminoethoxy)ethanol (D) to obtain the corresponding diclofenac *N*‐Boc‐2‐(2‐aminoethoxy)ethylester (E, step 2). Treatment of E with trifluoroacetic acid (TFA) generated the corresponding di‐TFA salt of diclofenac 2‐(2‐aminoethoxy)ethyl ester (F, step 3). Compounds C and F were then coupled to get SYN321 (step 4). During the last step of the synthesis, compound F can be coupled to any of both succinate moieties of compound C (position 2 of the glucuronic acid and/or position 6 of the glucosamine).

### SYN321 – Hydrolysis Profile

4 µL of 100 µg mL^−1^ stock solution in 50% Dimethyl sulfoxide (DMSO) of SYN321 was added to 396 µL of mixed gender plasma or synovial fluid to obtain 1 µg mL^−1^ incubation concentrations. Spiked study matrixes were incubated for 48 h with continuous shaking at 600 rpm 37 °C and samples were taken at 0, 4, 6, 24, and 48 h time points. Samples were quenched after collecting by precipitating with twofold volume of acetonitrile containing 100 mg mL^−1^ warfarin as internal standard (50 µL sample + 100 µL of precipitation solution with internal standard), then stored at −20 °C until analysis. For analysis, the precipitated samples were centrifuged for 10 min at 2272 x g at room temperature. Supernatant was transferred to analytical 96‐well plate and submitted for liquid chromatography–mass spectrometry (LC‐MS) analysis. The LC‐MS method for analysis and quantitation of diclofenac (1), diclofenac lactam (2) and linker (3) are shown in Tables S1 and S2 (Supporting Information). The standard samples were prepared in human plasma and synovial fluid, by spiking the matrix to concentrations of 10 – 50 000 nM of compounds 1, 2, and 3 using one volume of spiking solution (50% DMSO) and nine volumes of matrix, otherwise preparing them for analysis like samples.

The method performance parameters of compounds 1, 2, and 3 quantitation in human plasma are shown in Tables S3 and S4 (Supporting Information). Quantitation range for compounds 1 and 2 was 10– 50 000 ng mL^−1^ in plasma and synovial fluid, and for compound 3 10– 50 000 and 100 – 50 000 ng mL^−1^ in plasma and synovial fluid, respectively.

### Evaluation of Diclofenac Release in the uBeat^®^ MultiCompress Platform

Diclofenac release in culture medium caused by the hydrolysis of SYN321 ester bonds was further tested inside the uBeat MultiCompress platform, both in static and dynamic (HPC) conditions. Two different experimental setups were considered: i) Setup 1, where both channels 2 and 4 were injected with cell‐laden fibrin and ii) Setup 2, where only channel 4 was injected with cell‐laden fibrin whereas channel 2 was loaded with fibrin only. hACs were expanded, harvested and embedded in fibrin gel and cultured in chondrogenic medium within uBeat MultiCompress platform for 14 days as previously described. Subsequently, chondrogenic medium was removed, SYN321 was injected in channel 3. The corresponding reservoir at the end of channel 3 was also filled with SYN321, to prevent evaporation and/or diclofenac dilution, which would have otherwise happened if left unfilled or if filled with culture medium, respectively. Medium reservoirs were filled with 40 µL of serum free medium each. Two negative control configurations were prepared: i) both channel 2 and 4 injected with cell‐laden fibrin but no SYN321 loaded in channel 3 and ii) fibrin with no cells loaded in channel 2 and 4 and SYN321 loaded in channel 3. For each condition, half devices were cultured under HPC and the other half in static conditions for three days. In addition, technical conditions were added to measure the potential absorption of diclofenac to PDMS or its binding to culture medium, by dissolving diclofenac either in DMEM or in serum free medium. To calculate the concentration of diclofenac in such controls, the maximum amount of diclofenac potentially released by SYN321 in uBeat MultiCompress platform was computed. Since SYN321 dry powder contains 8% of diclofenac and SYN321 was used at a concentration of 15 mg mL^−1^, a maximum of 1.2 mg of diclofenac can be released in 1 mL (i.e., in the hypothesis that all diclofenac is released). Since that in one device a total of 1.8 µL of SYN321 (i.e., 0.6 µL per chamber) is injected, a maximum of 2.16 µg of diclofenac can be released. Final considerations on the total volume in the device (0.48 mL of serum free medium per device, i.e., 160 µL per chamber given by 40 µL per reservoir, excluding the SYN321 dedicated reservoirs), yield to a maximum final concentration of diclofenac in the medium equal to 4.5 µg mL^−1^. This concentration was considered for the additional technical control conditions. To this aim, sodium diclofenac powder (Sigma Aldrich) was dissolved first in DMSO used as vehicle (1:10 000), and subsequently in the final solutions, to reach a final concentration of 4.5 µg mL^−1^, assuming a 100% quantity of diclofenac in the powder. In particular, three technical conditions were prepared: i) MultiCompress platform filled with diclofenac 4.5 µg mL^−1^ dissolved in serum free medium, to detect a potential interaction with the PDMS device; ii) diclofenac 4.5 µg mL^−1^ dissolved in serum free medium to detect potential binding of the diclofenac to serum free medium; iii) diclofenac 4.5 µg mL^−1^ dissolved in DMEM containing 10 mM Hepes, 1 mM sodium pyruvate, 100 U mL^−1^ penicillin, 100 µg mL^−1^ streptomycin, 0.292 mg mL^−1^ L‐glutamine, to detect potential binding to medium. For each condition, half devices were cultured under HPC and the other half in static conditions for three days. After that, medium was collected from each device and stored at −80 °C for mass spectrometry analysis as described below.

### Rat MIA Model

Animals were anesthetized using Ketamine Xylazine mixture on study day 0. Then, 50 µL of MIA prepared at a concentration of 60 mg mL^−1^ in saline (i.e., 3 mg of MIA) was injected into the space between the tibia and femur in the flexed right knee joint using 30G needle. Subsequently, on day 11 of the study, different interventions were administered to the respective groups (Table 3), as follows: (i) in Group 1 (saline per vehicle), animals received a saline injection into the same knee; (ii) in Group 2 (HA), animals were treated with HA injected into the knee and diclofenac was administered orally once on the same day; (ii) in Groups 3–5 (SYN321), animals were injected with SYN321 into the right knee. The specific details of SYN321 treatments varied among groups 3, 4, and 5. In details, saline vehicle was supplied as ready to use (0.9% NaCl, Baxter) and each animal in group 1 was injected with 50 µl saline, via IA injection, into the right knee joint using 30G needle once on study day 11. For group 2, HA at a concentration of 10 mg mL^−1^ in saline was prepared by weighing out 10 mg of HA in a closed container, transferring 1 mL of saline solution to the container, and shaking the sample on a shakeboard for 10–12 h (100‐200 rotations min^−1^). On study day 11, every animal in group 2 received a 50 µL injection of HA into their right knee joint through intra‐articular injection, administered with a 30G needle. Moreover, 8 mL saline were added to an ampoule of 2 mL diclofenac at a concentration of 25 mg mL^−1^ (Teva) and vortexed to obtain a final diclofenac concentration of 5 mg mL^−1^. Animals in group 2 weighing 200 g were dosed with 1 mL diclofenac (i.e., dose volume of 5 mL kg^−1^, dose level of 25 mg kg^−1^) per os (PO) once on study day 11.

For group 3, SYN321 at a concentration of 10 mg mL^−1^ in saline was prepared by weighing out 10 mg of SYN321, transferring 1 mL of saline solution, and shaking the sample on a shakeboard for 10–12 h (100‐200 rotations min^−1^). The solution was kept in the fridge. Each animal was injected with 50 µL (i.e., 0.5 mg) of SYN321, via intra‐articular injection, once on study day 11. For group 4, a concentration of 3 mg mL^−1^ SYN321 was used. Specifically, 100 uL (100 mg) of the 10 mg mL^−1^ stock solution of SYN321 was transferred to a vial and diluted with 233 uL saline solution. The vial was shaken for 1 min and allowed to stand for 1 h before shaken again for 1 min. The process was repeated 4 times and the solution which should be homogenous was inspected visually before using it. On study day 11, each animal underwent a single intra‐articular injection of 50 µl (0.15 mg) of SYN321 into their right knee joint. Finally, SYN321 at a concentration of 1 mg mL^−1^ was administered to group 5, as follows. 100 µL (100 mg) of the 10 mg mL^−1^ stock solution of SYN321 was transferred to a vial and diluted with 900 µL Saline solution. The vial was shaken as described above to obtain a homogeneous solution. On the 11th day of the study, a 30G needle was used to administer a single IA injection of 50 µL (0.15 mg) of SYN321 into the right knee joint of each animal.

### Body Weights

Animals’ body weight was measured at the beginning of the study (day −1, baseline) and every 4 days, starting from day 11 (i.e., 11, 15, 19, 23, 28, 32, 36, 40, and 44).

### Weight Bearing Test

On specific time‐points, incapacitance tests were performed on rats. The animal was located in a holder specially designed to maintain the animal comfortably positioned on two separated sensor plates. The incapacitance device enabled then to quantify the spontaneous postural changes reflecting spontaneous pain, by independently measuring the weight that the animal applies to each hind paw on two separate sensors. Since normal rat distributes weight equally on both paws, change of this equilibrium can reflect the level of discomfort due to an injured paw. On testing day, each rat was placed in the test apparatus, and three measurements were recorded through the incapacitance meter over a period of 5 s. These values were then averaged. Weight bearing deficits were measured for all rats on study day −1 (baseline), then on study days 10, 12, 14, 17, and then once weekly. Each treatment group was compared to the vehicle group using appropriate statistical test.

To reduce variability and increase the likelihood of a successful study, on day −2, each rat was placed in the test apparatus (incapacitance meter) for 5–10 min (i.e., habituation to test apparatus).

### Open‐Field Test

On study day 10, animals were placed in the open‐field apparatus for a period of 5 min and their pretreatment level was recorded. A computerized apparatus monitored the speed and distance that the animals walked during this period. Open‐Field was then recorded on study days 24 and 45, following the Weight bearing test.

### Plasma and Synovial Fluid Analysis

Plasma samples were collected from all animals starting at day 12 and subsequently at 18, 25, 32, 39, and 49 days. Animals were slightly anesthetized with CO_2_ and bled at retro orbital sinus. 100 µL of whole blood samples were collected in EDTA K3 tubes and centrifuged at 3000 rpm for 5 min. The plasma was collected using filtered pipette tips and the vials containing the plasma were snap frozen in liquid nitrogen and stored at −80 °C. Using a reverse phase gradient HPLC and subsequently detecting them using positive electrospray ionization and multiple reaction monitoring (MRM), all 282 rat plasma samples were analyzed for diclofenac (1) and linker (3).

At study termination, synovial fluid was collected from all animals for analysis of inflammatory cytokines (MCP‐1, IL‐6, KC/GRO and MIP‐3α) using Milliplex Rat Cytokine/Chemokine Magnetic Bead Panel Kit (Millipore).

### Evaluation of SYN321 Efficacy in the uBeat® MultiCompress Platform

SYN321 was used as first case study for the validation of the microfluidic platform with a therapeutic product under development. hACs were expanded, harvested and embedded in fibrin gel and cultured in chondrogenic medium for 14 days as previously described. After that, some devices were kept in culture under static conditions as “healthy controls” up to 21 days, while the remaining ones were subjected to HPC for 7 days. After that, SYN321 was injected in channel 3, while two additional positive controls were added: i) NaHA 15 mg mL^−1^, that was injected as hydrogel in channel 3; ii) Serum free medium with dissolved diclofenac at a concentration of 4.18 µg mL^−1^, that was injected in channel 3. In all the devices, chondrogenic medium in lateral channels was replaced with serum free medium. Devices were then cultured for 3 days under HPC, except for the “healthy controls”. After three days, samples were collected for RT‐qPCR and immunofluorescence as described below.

### Mass Spectrometry Analysis

Mass Spectrometry Analysis was performed by Recipharm OT Chemistry on the medium samples collected by BiomimX to measure the diclofenac release from SYN321 cultured in the microfluidic platform. Stock solutions of diclofenac (Sigma Aldrich) (i.e., diclofenac‐*d_4_*) were prepared at the concentration of 1.00 mg mL^−1^ in DMSO which was used for the preparation of the calibration samples in Acetonitrile (ACN) containing internal standard (IS). diclofenac‐*d_4_* solution at 1.00 mg mL^−1^ was used to prepare the 20.0 ng mL^−1^ IS solution in ACN. Calibration standards (Cal 1 to Cal 7) used for the creation of the calibration curve were prepared, in ACN containing the IS at 20 ng mL^−1^, using the stock solution of diclofenac. Lower concentration calibration standards were prepared by serial dilution of higher calibration standards. Calibration samples were prepared with 20.0 µL of blank DMEM (Life Technologies Ltd.) and 60.0 µL of calibration standard (Cal 1 to Cal 7) containing IS that were pipetted in a 384 well plate. Two sets of calibration samples were prepared. Blank samples and Blank with IS samples were prepared with 20.0 µL of blank DMEM and 60.0 µL of ACN that were pipetted in a 384 well plate.20.0 µL of blank DMEM and 60.0 µL of ACN with IS were pipetted in a 384 well plate. Two sets of blank and blank with IS samples were prepared. The target samples (i.e., from BiomimX study) were prepared with 20.0 µL of medium from the study samples and 60.0 µL of ACN with IS were added into a 384 well plate. After mixing, all samples (blanks, calibration curve samples, and study samples) were centrifuged for 10 min (4000 RPM) at room temperature. The supernatants of all samples were analyzed by the Ultra‐Performance Liquid Chromatography coupled with Tandem Mass Spectrometry (UPLC‐MS/MS) system. During analysis, a set of a calibration samples was analyzed before and after the qualification samples. Integrations, calibrations and calculations were performed using TargetLynx XS as well as Office 365.

### Immunofluorescence

Immunofluorescence analyses were performed on cartilage models at day 14, 21, and 23. Medium channels were washed with PBS, then the microtissues were fixed in 4% paraformaldehyde at room temperature for 30 min. The devices were disassembled by removing the glass coverslip and by peeling off the PDMS actuation compartment, to expose the microtissues. Cells were permeabilized with 0.1% Tween‐20 (Sigma Aldrich) and a blocking solution with 5% Goat Serum (Sigma Aldrich) was applied for 1 h at room temperature to block non‐specific bindings. Samples were incubated for 16 h at 4 °C with a Primary antibody solution. Mouse anti‐human IgG1 Aggrecan (dilution 1:200; Santa Cruz Biotech.) was used to evaluate cartilage maturation at day 14. Mouse anti‐human IgG1 Aggrecan and Rabbit anti‐human IgG MMP13 (dilution 1:200; Invitrogen) were used to assess the effects of HPC on the cartilage model (day 21), as well as the effect of therapeutic formulations on HPC‐induced OA microtissues (day 23). Secondary Antibodies Alexa Fluor 546 Goat anti‐mouse IgG (H+L) and Alexa Fluor Plus 488 Goat anti‐rabbit IgG (H+L) were then supplied for 6 hours at 4 °C at 1:200 dilution, together with 4′,6‐diamidino‐2‐phenylindole dihydrochloride 300 nM (DAPI, Thermofisher), that was used to identify cell nuclei. Representative images of three different regions for each microtissue were acquired using fluorescence microscope (Olympus IX83) and analyzed using ImageJ software. Three microtissues for each condition were considered for the immunofluorescence analysis.

### RT‐qPCR

Quantification of gene expression was performed by RT‐qPCR analysis. This analysis was carried out in three stages.

Medium channels were washed with PBS and the devices were opened to expose the microtissues, that were collected in a buffer solution. RNA extraction was then performed using NORGEN Total RNA Purification Micro Kit (cat. 35300). and RNA concentration was quantified using NanoDrop (Thermofisher). The RNA samples were retro transcribed into cDNA using Superscript Reverse Transcriptase (cat. 18080‐044, ThermoFisher). The cDNA samples were diluted to reach the concentration of 5 ng µL^−1^ and RT‐qPCR was performed to quantify the expression of the following genes: COL1A1 (Hs00164004_m1), COL2A1 (Hs00264051_m1), COL10A1 (Hs00166657_m1), MMP13 (Hs00233992_m1), ACAN (Hs00153936_m1), PRG4 (Hs00981633_m1), IL6 (Hs00985639_m1), COX‐2 (Hs00153133_m1), TNF‐α (Hs01113624_g1), PIEZO1 (Hs00207230_m1), PIEZO2 (Hs00926218_m1), TRPV4 (Hs01099348_m1). Glyceraldehyde 3‐phosphate dehydrogenase (GAPDH, Hs02758991_g1) housekeeping gene was used as reference. For each condition, n≥3 biologically independent samples were considered.

### Statistical Analysis

The results of RT‐qPCR are represented as means + SD. Statistical analysis was performed using GraphPad Prism 8. Normally distributed data populations were assessed using Shapiro‐Wilk test. Outliers were removed according to ROUT method (Q = 1%). Mann‐Whitney U‐test was used when comparing two non‐normal distributed populations. Multiple comparisons were done by using ordinary one‐way analysis of variance (ANOVA). Non‐normally distributed variables were compared using Kruskal‐Wallis test with Dunn's multiple comparison test. Statistical significance was indicated by * P<0.05 and ** P<0.01.

### Animal Ethical Statement

The animal study has been performed following approval of an application form submitted to the Committee for Ethical Conduct in the Care and Use of Laboratory Animals that stated that the study complied with the rules and regulations set forth (*IACUC license number IL‐20‐6‐239*).

## Conflict of Interest

Roberta Visone, Marco Rasponi, and Paola Occhetta share equities in BiomimX Srl. Rune Ringom and Ulf Björklund own shares in Synartro AB.

## Author Contributions

C.P. and S.P. contributed equally to this work. P.O., M.R., and R.V. conceived the project. P.O., M.R., R.V., and S.P. conceived the device. C.P. and S.P. produced the molds and the devices. S.P. performed the biological experiments in vitro. C.P. and S.P. analyzed the results of the biological experiments in vitro. U.B. supervised and designed the in vivo experiments. A.B.G. and R. R. conceived and supervised the in vitro release experiments. R.R. performed and supervised the synthesis and analysis conducted at Recipharm OT. C.P., S.P., A.B.G., and P.O. wrote the manuscript. All authors discussed the results, commented on the manuscript, and contributed to its final version.

## Supporting information



Supporting Information

## Data Availability

The data that support the findings of this study are available from the corresponding author upon reasonable request.
