# Supplementary material for: An Advanced Mechanically Active Osteoarthritis‐on‐Chip Model to Test Injectable Therapeutic Formulations: The SYN321 Case Study
Source: Adv Healthc Mater. 2024 Sep 24;13(32):2401187. doi: 10.1002/adhm.202401187 (PMC11670293; doi:10.1002/adhm.202401187)
Supplement: Supplementary file 1 — Supporting Information [file ADHM-13-0-s001.pdf]

ADVANCED HEALTHCARE MATERIALS

Supporting Information

for *Adv. Healthcare Mater.*, DOI 10.1002/adhm.202401187

An Advanced Mechanically Active Osteoarthritis-on-Chip Model to Test Injectable Therapeutic Formulations: The SYN321 Case Study

*Cecilia Palma, Stefano Piazza, Roberta Visone, Rune Ringom, Ulf Björklund, Antonio Bermejo Gómez, Marco Rasponi and Paola Occhetta**

SUPPORTING INFORMATION

An advanced mechanically active osteoarthritis-on-chip model to test injectable therapeutic formulations: the SYN321 case study

Cecilia Palma[*,1], Stefano Piazza[*,2], Roberta Visone[2], Rune Ringom[3], Ulf Björklund[4], Antonio Bermejo Gómez[5], Marco Rasponi[1], Paola Occhetta[1,2]

*Equally contributing authors

C. Palma, M. Rasponi, P. Occhetta
Department of Electronics, Information and Bioengineering
Politecnico di Milano
Via Ponzio 34/5 – 20133 Milan, Italy
E-mail: paola.occhetta@polimi.it, marco.rasponi@polimi.it

S. Piazza, P. Occhetta
BiomimX Srl
Viale Decumano 41, MIND - Milano Innovation District – 20157 Milan, Italy

R. Ringom
Recipharm OT Chemistry AB
Virdings allé 18, 754 50 Uppsala, Sweden

U. Björklund
UB-consulting AB
Trädgårdsgatan 7A, 753 09 Uppsala, Sweden

A. Bermejo Gómez
Synartro AB
Dalgatan 16A, 752 18 Uppsala, Sweden

Hyaluronic acid (HA) description and molecular structure

Hyaluronic acid (HA) is an anionic, non-sulphated glycosaminoglycan distributed throughout connective, epithelial, and neural tissues in humans and in other vertebrates. It is a polysaccharide built of disaccharide repeating residues of β-D-glucuronic acid and *N*-acetyl-β-D-glucosamine, where the linkage is (1→3) from the glucuronic acid to the glucosamine, and (1→4) from the glucosamine to the glucuronic acid. Hyaluronan refers to all physiological forms of hyaluronic acid, the most common being the sodium salt (sodium hyaluronate; NaHA). However, the term hyaluronic acid is commonly used in the literature for referring to any of its forms.

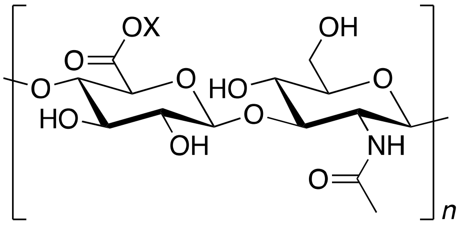

Hyaluronic acid (X = H)
Sodium hyaluronate (X = Na)

Figure S1. Structure of hyaluronic acid (HA) and sodium hyaluronate (NaHA)

SYN321 – Synthesis

The synthesis of SYN321 followed the protocol outlined in EP 3 226 905 81 (entitled '*Hyaluronan conjugates with pharmaceutically active substances, methods and compositions*'). In details, the procedure involved the subsequent steps:

i. Synthesis of [2-(2,6-dichloro-phenylamino)-phenyl]-acetic acid 2-(2-tert-butoxycarbonylamino-ethoxy)-ethyl ester (E):
Diclofenac (1.2 g), [2-(2-hydroxy-ethoxy)-ethyl]-carbamic acid tert-butyl ester (1,4 g) and 4-dimethylamino- pyridine (DMAP) (76 mg) were dissolved in dichloromethane (DCM) (6 mL). The reaction mixture was cooled on an ice- water bath, 1-ethyl-3-(3-dimethylaminopropyl)carbodiimide (EDC) (1.31 g) was added and was stirred for 4 h while the ice was melting. LCMS of the reaction mixture showed that the expected product was formed and no starting material was left. The reaction mixture was transferred to a separation funnel and about 7 mL of DCM was added. The DCM phase was washed with water (~3*10 mL) and the DCM phase was evaporated. Obtained 2 g crude material. Flash chromatography in EtOAc/Heptane 1/1 gave 1.166 g.

ii. Synthesis of [2-(2,6-dichloro-phenylamino)-phenyl]-acetic acid 2-(2-amino-ethoxy)-

ethyl ester (F):

[2-(2,6-Dichloro-phenylamino)-phenyl]-acetic acid 2-(2-tert-butoxycarbonylamino-ethoxy)-ethyl ester (1166 mg) was dissolved in DCM (9 mL). Trifluoroacetic acid (TFA) (1 mL) was added. The reaction mixture was heated at 40 °C for 15 min. [2-(2,6-Dichloro-phenylamino)-phenyl]-acetic acid 2-(2-amino-ethoxy)-ethyl ester was obtained after evaporation as the ditrifluoroacetate salt, 1.47 g.

iii. Synthesis of hyaluronan succinyl ester (C):

Sodium hyaluronate (1000 mg) was dissolved in formamide (100 mL). Pyridine (Py) (2014 mL), DMAP (30 mg) and succinic anhydride (2494 mg) were added. The reaction mixture was stirred at Room temperature. The reaction mixture was dialyzed in water for 24 hours. The reaction mixture was dialyzed in 1% NaCl for 24 h. The product was precipitated in ethanol (1 L), collected and dried in vacuum overnight. Hyaluronan succinyl ester was obtained 1.043 g.

iv. Synthesis of SYN321:

Hyaluronan succinyl ester (200 mg) was dissolved in water (5 mL). DMF (15 mL) was added to obtain a 30 solution of succinyl hyaluronan in water/DMF, 1/3 (20 mL). *N*-methylmorpholine (33 mL), HOBT (0,5 mg) and a DMF solution of [2-(2,6-dichloro-phenylamino)-phenyl]-acetic acid 2-(2-amino-ethoxy)-ethyl ester 397 mg/mL (46,4 mL, 18,4 mg) were added to the stirred succinyl-hyaluronan solution. EDC (5.8 mg) was added. The reaction mixture was mixed thoroughly and left over night at room temperature. Sodium chloride (200 mg) was added as a sat solution (359 mg/mL) to the DMF-water solution. The product was precipitated in ethanol (100 ml) and stirred for 2.5 h. The precipitate was 35 collected and dissolved in 1% NaCl (20 mL) and precipitated in ethanol (80 mL). The solid material was collected, and once more dissolved in 1% NaCl and precipitated. The precipitate was dissolved in water and lyophilized. Obtained 177 mg. According to proton ^{1}H-NMR spectroscopy the degree of substitution was 0.22.

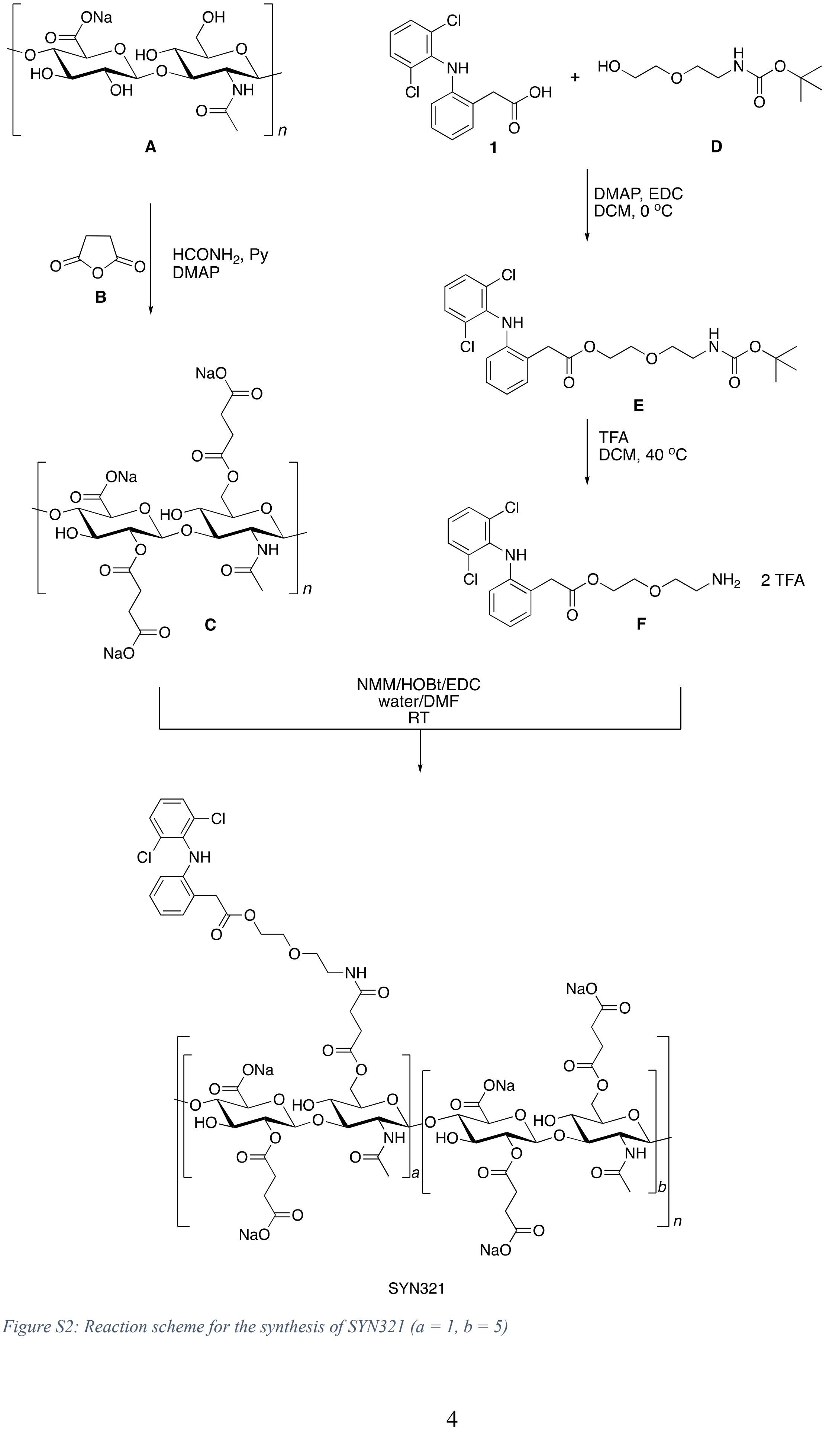

Figure S2: Reaction scheme for the synthesis of SYN321 (a = 1, b = 5)

Evaporation within the uBeat® MultiCompress platform

To test the evaporation rate within the uBeat® MultiCompress platform, three devices were injected with fibrin gel only (i.e., final concentration 2 U/ml Thrombin, 10mg/ml fibrinogen) and cultured at 37°C 5%CO2 over weekend with PBS in channels 1-3-5. Then, using Bovine Serum Albumin (BSA) from Pierce BCA Protein Assay Kit, ThermoFisher, a solution of 0.5mg/ml of BSA in PBS was prepared and 200 µl were placed in the channels 1-3-5 and the corresponding reservoirs of each chamber of the devices. After three hours, BCA solution was collected from each chamber of one device and stored at +4°C. The other two devices were kept in culture for a total of three days, one in static and one in dynamic conditions (2h of stimulation, 4h of rest, 2h of stimulation and 16h of rest) and then the BSA solution was collected from them. A BSA calibration scale was prepared (2mg/ml, 1mg/ml, 0.5mg/ml, 0.25mg/ml, 0.125mg/ml, 0.0625mg/ml, 0.03125mg/ml, 0mg/ml) and 25µl of scale's duplicates were transferred in a 96 multiwell plate together with 25µl of sample's duplicates. A solution of working reagent (WR) was prepared by mixing 50 parts of BCA reagent A with 1 part of BCA reagent B, then 200µl of WR were transferred in each well and the plate was incubated for 30 min at 37°C. Then, Absorbance at 550 nm was measured with Tecan spectrophotometer. Results were analysed in Excel. The blank value was subtracted from all the wells, a calibration curve was created by linear regression and samples' concentrations were obtained. Values were plotted using GraphPad Prism. Statistical analysis was performed using nonparametric Kruskal-Wallis test. As illustrated in the figure below, the BSA concentration in PBS increased after three days of static cultures (T2) within the uBeat® MultiCompress platform, compared to samples maintained for only three hours (T0). This suggests that some evaporation occurs within the device. Additionally, a higher BSA concentration was observed at T2 in dynamic samples compared to static samples, indicating that mechanical actuation further promotes evaporation.

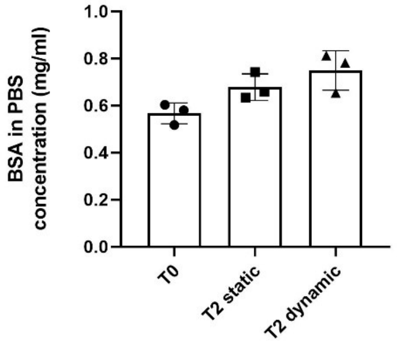

Figure S3: BSA concentration in PBS over time within the uBeat® MultiCompress platform comparing T0 (i.e. 3 hours in static conditions), T2 static (i.e. 3 days in static conditions) and T2 dynamic (i.e. 3 days in dynamic conditions). N=3.

SYN321 hydrolysis in synovial fluid and human plasma

Table S1. LC-MS method for analysis and quantitation of diclofenac (1).

Instrumentation	Waters Acquity UPLC + Thermo Q-Exactive Focus Orbitrap MS			
Column	Waters Acquity HSS T3 (2.1 × 50 mm, 1.8 µm) column with pre-column filter			
Ionization mode<br>Sheath gas<br>Auxiliary gas<br>Sweep gas<br>Capillary voltage<br>Capillary temperature<br>Auxiliary gas temperature<br>Mass range<br>RF lens<br>Resolution<br>Normalized collision energy<br>Calibration	ESI-<br>nitrogen 50 (Arbitrary units)<br>nitrogen 10 (Arbitrary units)<br>nitrogen 3 (Arbitrary units)<br>3200 V<br>320 °C<br>500 °C<br>m/z 100 – 600<br>80<br>70 000 (FWHM @ m/z 200) for full scan<br>Off for full scan<br>External			
Software	Thermo Xcalibur 4.1.31.9			
Other information	First 0.5 min of the run was directed into waste by using a divert valve to decrease the ion source contamination by early eluting matrix constituents.			
Gradient Elution; A = 0.1% acetic acid, B = 85:15 acetonitrile / isopropanol (v/v)				
Time (min)	Flow (ml/min)	A%	B%	curve
0.0	0.600	100	0	-
0.5	0.600	100	0	6
2.0	0.600	80	20	6
3.0	0.600	10	90	6
4.0	0.600	2	98	6
5.0	0.600	100	0	1
Column oven temperature 60 °C				
Injection volume 4 µl				
Ion chromatograms were extracted from the Q-Exactive Focus Orbitrap MS total ion chromatograms using calculated monoisotopic accurate masses for deprotonated molecular ions with 10 ppm window.				
Compound	m/z	Retention time (min)	Polarity	
Diclofenac	294.0094	3.05	ESI-	
Warfarin (IS)	307.0976	2.96	ESI-	

Table S2. LC-MS method for analysis and quantitation of diclofenac lactam (2) and linker (3).

Instrumentation	Waters Acquity UPLC + Thermo O-Exploris Orbitrap MS
Column	Waters Atlantis premier BEH C18 AX (2.1 × 100 mm, 1.7 µm) column with pre-column filter
Ionization mode<br>Sheath gas<br>Auxiliary gas<br>Sweep gas<br>Capillary voltage<br>Capillary temperature<br>Auxiliary gas temperature<br>Mass range<br>RF lens<br>Resolution<br>Calibration	ESI+<br>nitrogen 50 (Arbitrary units)<br>nitrogen 10 (Arbitrary units)<br>nitrogen 3 (Arbitrary units)<br>3000 V<br>320 °C<br>450 °C<br>m/z 100 – 600<br>80<br>120 000 (FWHM @ m/z 200) for full scan<br>External
Software	Thermo Xcalibur 4.1.31.9
Other information	First 0.5 min of the run was directed into waste by using a divert valve to decrease the ion source contamination by early eluting matrix constituents.

Gradient Elution; A = 2 mM ammonium formate, B = 85:15 acetonitrile / isopropanol (v/v)

Time (min)	Flow (ml/min)	A%	B%	curve
0.0	0.500	98	2	-
0.5	0.500	98	2	6
3.0	0.500	10	90	6
5.0	0.500	10	90	6
5.5	0.500	2	98	6
8.0	0.500	98	2	1

Column oven temperature 60 °C
Injection volume 4 µl

Ion chromatograms were extracted from the O-Exploris Orbitrap MS total ion chromatograms using calculated monoisotopic accurate masses for deprotonated molecular ions with 10 ppm window.

Compound	m/z	Retention time (min)	Polarity
Lactam	278.0134	3.00	ESI+
Linker	206.1023	1.54	ESI+
Warfarin (IS)	309.1121	3.25	ESI+

Table S3. Method performance of compounds quantitation in human plasma.

**Matrix**	Human plasma		
**Compound**	Diclofenac (1)	Lactam (2)	Linker (3)
**Detection limit (nM)**	10	10	10
**Quantitation limit (nM)**	10	10	10
**Range (nM)**	10 - 50 000	10 - 1 000	10 - 1 000
**R^2**	>0.999	0.996	>0.999
**Concentration (nM)**	**Accuracy % (n=2)**	**Accuracy % (n=2)**	**Accuracy % (n=2)**
10	121.8	100.0	100.0
100	86.4	100.0	100.0
1000	90.5	100.0	100.0
10000	101.3	-	-
50000	100.0	-	-
**Snedecor precision (%)***	17.4	14.7	9.5
**Calibration curve fitting**	Quadratic fitting, weighting 1/X		
**Internal standard**	Warfarin		

Table S4. Method performance of compounds quantitation in human synovial fluid.

**Matrix**	Human synovial fluid					
**Compound**	Diclofenac (1)		Lactam (2)		Linker (3)	
**Detection limit (ng/ml)**	10		10		100	
**Quantitation limit (ng/ml)**	10		10		100	
**Range (ng/ml)**	10 - 50 000		10 - 50 000		100 - 50 000	
**R^2**	> 0.999		0.993		0.986	
**Conc. (ng/ml)**	**Acc. % (n=3)**	**Prec. % (n=3)**	**Acc. % (n=3)**	**Prec. % (n=3)**	**Acc. % (n=3)**	**Prec. % (n=3)**
10	105.0	14.5	117.7	15.3	-	-
100	97.3	8.6	86.0	18.8	75.6	14.9
1 000	97.4	0.9	95.5	19.4	127.8	20.2
10 000	100.3	2.0	100.8	16.6	96.6	16.4
50 000	100.0	1.8	100.1	9.1	96.4	15.4
**Calibration curve fitting**	Quadratic, weighting 1/X					
**Internal standard**	Warfarin					

Effect of SYN321 in a rat MIA model of osteoarthritis: tables reporting results of body weight, Weight Bearing tests and distance

Table S5: Mean group body weight (g).

	Group 1		Group 2		Group 3		Group 4		Group 5	
**Days**	**Mean**	**SEM**	**Mean**	**SEM**	**Mean**	**SEM**	**Mean**	**SEM**	**Mean**	**SEM**
Baseline	241.00	3.13	239.40	3.13	243.00	4.11	235.10	2.17	237.20	2.48
11	300.40	4.20	292.10	3.80	305.10	5.05	295.90	2.75	294.40	4.50
15	318.78	5.97	295.20	8.84	320.00	6.21	311.89	3.19	313.30	5.65
19	341.11	6.62	324.30	4.76	338.78	8.28	334.11	3.47	336.00	6.18
23	369.00	8.02	346.00	5.98	369.22	8.39	357.44	4.65	357.80	7.42
28	382.33	8.92	357.80	6.22	371.00	8.87	369.44	5.11	367.30	8.79
32	395.11	9.42	369.70	6.79	384.44	9.25	382.00	5.19	379.10	9.45
36	403.56	11.86	375.44	7.79	390.78	9.34	394.24	4.84	384.40	10.71
40	414.22	12.30	384.22	8.12	399.56	9.15	402.78	4.90	392.20	11.99
44	432.33	12.62	400.63	10.24	415.33	9.48	414.89	5.65	406.40	12.29

Table S6: Mean group body weight (% from baseline).

	Group 1		Group 2		Group 3		Group 4		Group 5	
**Days**	**Mean**	**SEM**	**Mean**	**SEM**	**Mean**	**SEM**	**Mean**	**SEM**	**Mean**	**SEM**
Baseline	100.00	0.00	100.00	0.00	100.00	0.00	100.00	0.00	100.00	0.00
11	124.65	0.77	122.13	1.78	125.58	0.75	125.88	0.74	124.10	1.23
15	132.77	1.04	123.51	3.95	131.67	1.05	133.18	0.97	132.01	1.22
19	142.06	1.24	135.50	1.44	140.10	1.54	142.67	1.11	141.57	1.38
23	153.65	1.67	144.53	1.76	152.80	2.34	152.60	1.29	150.72	1.78
28	159.17	1.97	149.47	1.91	153.52	2.43	157.74	1.67	154.74	2.65
32	164.50	2.21	154.43	2.05	159.07	2.44	163.08	1.45	159.71	2.98
36	167.90	2.97	156.63	2.16	161.72	2.66	168.34	1.75	161.92	3.53
40	172.34	3.17	160.30	2.42	165.34	2.40	172.00	1.82	165.19	4.09
44	179.88	3.18	167.25	3.10	171.94	3.08	177.22	2.63	171.16	4.10

Table S7: Mean group Weight Bearing (difference between legs) (L%-R%).

	Group 1		Group 2		Group 3		Group 4		Group 5	
**Days**	**Mean**	**SEM**	**Mean**	**SEM**	**Mean**	**SEM**	**Mean**	**SEM**	**Mean**	**SEM**
Baseline	-0.29	0.58	-0.45	0.49	-0.44	0.63	-0.60	0.49	-0.15	0.57
10	17.83	0.85	17.00	1.30	19.24	1.12	13.87	0.71	17.24	0.80
12	22.39	2.48	18.12	2.28	11.60[a]	2.34	13.56	4.31	15.96	2.75
14	25.59	1.49	18.56	1.43	19.62	2.09	18.68	2.47	18.14	2.73
17	27.23	1.49	23.38	2.55	20.77	1.54	22.02	3.15	20.15	1.51
24	24.63	2.62	24.06	3.01	21.54	2.21	20.97	2.37	18.43	2.12
31	21.24	1.46	25.29	3.05	22.54	2.84	19.61	3.16	18.71	1.68
38	20.37	2.34	23.14	2.03	19.12	2.91	18.33	2.15	21.85	2.22
45	18.72	1.80	19.89	3.15	19.51	1.82	17.06	0.78	21.85	2.00

[a]$p<0.05$ vs. Vehicle (Group 1) using one way ANOVA followed by Dunnett's test.

Table S8: Mean group distance (meters).

	Group 1		Group 2		Group 3		Group 4		Group 5	
**Days**	**Mean**	**SEM**	**Mean**	**SEM**	**Mean**	**SEM**	**Mean**	**SEM**	**Mean**	**SEM**
10	29.97	1.33	31.30	1.71	28.75	1.30	20.65	3.85	26.54	2.67
24	35.96	1.93	25.95	2.85	25.97	4.15	30.76	3.12	27.83	4.25
38	24.19	2.16	13.07	2.66	17.20	2.49	19.95	2.02	12.81	3.47
